# Molecular Systematics of the Genus *Acidithiobacillus*: Insights into the Phylogenetic Structure and Diversification of the Taxon

**DOI:** 10.3389/fmicb.2017.00030

**Published:** 2017-01-19

**Authors:** Harold Nuñez, Ana Moya-Beltrán, Paulo C. Covarrubias, Francisco Issotta, Juan Pablo Cárdenas, Mónica González, Joaquín Atavales, Lillian G. Acuña, D. Barrie Johnson, Raquel Quatrini

**Affiliations:** ^1^Microbial Ecophysiology Laboratory, Fundación Ciencia & VidaSantiago, Chile; ^2^Faculty of Biological Sciences, Andres Bello UniversitySantiago, Chile; ^3^uBiome, Inc.San Francisco, CA, USA; ^4^College of Natural Sciences, Bangor UniversityBangor, UK

**Keywords:** *Acidithiobacillus*, species complex, phylogenetic structure, diversity, 16S rRNA, MLSA, targeted metagenomics

## Abstract

The acidithiobacilli are sulfur-oxidizing acidophilic bacteria that thrive in both natural and anthropogenic low pH environments. They contribute to processes that lead to the generation of acid rock drainage in several different geoclimatic contexts, and their properties have long been harnessed for the biotechnological processing of minerals. Presently, the genus is composed of seven validated species, described between 1922 and 2015: *Acidithiobacillus thiooxidans, A. ferrooxidans, A. albertensis, A. caldus, A. ferrivorans, A. ferridurans*, and *A. ferriphilus*. However, a large number of *Acidithiobacillus* strains and sequence clones have been obtained from a variety of ecological niches over the years, and many isolates are thought to vary in phenotypic properties and cognate genetic traits. Moreover, many isolates remain unclassified and several conflicting specific assignments muddle the picture from an evolutionary standpoint. Here we revise the phylogenetic relationships within this species complex and determine the phylogenetic species boundaries using three different typing approaches with varying degrees of resolution: 16S rRNA gene-based ribotyping, oligotyping, and multi-locus sequencing analysis (MLSA). To this end, the 580 16S rRNA gene sequences affiliated to the *Acidithiobacillus* spp. were collected from public and private databases and subjected to a comprehensive phylogenetic analysis. Oligotyping was used to profile high-entropy nucleotide positions and resolve meaningful differences between closely related strains at the 16S rRNA gene level. Due to its greater discriminatory power, MLSA was used as a proxy for genome-wide divergence in a smaller but representative set of strains. Results obtained indicate that there is still considerable unexplored diversity within this genus. At least six new lineages or phylotypes, supported by the different methods used herein, are evident within the *Acidithiobacillus* species complex. Although the diagnostic characteristics of these subgroups of strains are as yet unresolved, correlations to specific metadata hint to the mechanisms behind econiche-driven divergence of some of the species/phylotypes identified. The emerging phylogenetic structure for the genus outlined in this study can be used to guide isolate selection for future population genomics and evolutionary studies in this important acidophile model.

## Introduction

The genus *Acidithiobacillus* (Kelly and Wood, 2000), recently assigned to a new class, *Acidithiobacillia*, of the phylum Proteobacteria (Williams and Kelly, 2013) includes species of Gram-negative, rod-shaped, autotrophic bacteria that are non-sporulating, obligate acidophiles, and catalyze the dissimilatory oxidation of elemental sulfur and reduced inorganic sulfur compounds (Garrity et al., 2005). For many years, this group of bacteria has been exploited in the bioleaching of metal sulfides, the desulfurization of coal, and natural gas, among other uses (Johnson, 2014). Representatives of *Acidithiobacillus* occur world-wide in a diverse range of natural (acid rock drainage, sulfur springs, etc.) and industrial settings (ore concentrates, pulps, and leaching solutions of the mining industry, etc.), with varying physicochemical characteristics (e.g., redox potentials and concentrations of dissolved solutes). A large number of strains living in these various ecological niches have been described over the years, and more recently also a vast number of sequence clones have been deposited in public databases, spanning a great deal of the inherent diversity of this taxon (Nuñez et al., 2016).

Until relatively recently, the *Acidithiobacillus* genus comprised only four validated species: *A*. *thiooxidans* (Waksman and Joffe, 1922), *A. ferrooxidans* (Temple and Colmer, 1951), *A. albertensis* (Bryant et al., 1983), and *A. caldus* (Hallberg and Lindström, 1994), with all isolates that could oxidize ferrous iron as well as reduced sulfur being considered as strains of *A. ferrooxidans*. However, in the last two decades, a variety of molecular tools suited for typing and identification of bacteria have been applied to further revise the taxon (reviewed in Nuñez et al., 2016). *Acidithiobacillus* strains of diverse origins have since been assigned to distinct phylogenetic subgroups and/or genomovars, thought to represent a number of unidentified cryptic species (e.g., Luo et al., 2009; Amouric et al., 2011; Wu et al., 2014). Based on a careful re-evaluation of phenotypic characteristics (e.g., capacity to oxidize molecular hydrogen, temperature, and pH tolerance profiles, tolerance to elevated concentrations of transition metals and chloride, presence of flagella, etc.) and multilocus sequence analyses, three novel iron-oxidizing species have been recently recognized, *Acidithiobacillus ferrivorans* (Hallberg et al., 2010), *Acidithiobacillus ferridurans* (Hedrich and Johnson, 2013), and *Acidithiobacillus ferriphilus* (Falagán and Johnson, 2016), enlarging the genus to a current total of seven species. Provisional recognition of a number of additional (*Acidi*)*thiobacillus* species—associated to particular niches—has occurred in the past, e.g., “(*Acidi)thiobacilus concretivorus,”* the predominant isolate during the acidification associated to the final stage of concrete corrosion (Parker, 1945a,b, 1947) and “*Acidithiobacillus cuprithermicus,”* described as a novel isolate growing on chalcopyrite obtained from the Tinto River (Fernández-Remolar et al., 2003), though the validity of some proposed novel “species” has often been questioned (e.g., Vishniac and Santer, 1957).

During the last decade, several hundreds of *Acidithiobacillus* strains have been isolated from all over the world (e.g., Ni et al., 2008) and a large number of 16S rRNA sequence clones have been obtained from environmental studies (summarized in Huang et al., 2016). Evidence is beginning to accumulate that supports both spatial and temporal variations in the occurrence and distribution of *Acidithiobacillus* species types that dominate acidophilic prokaryotic communities from different environments (e.g., Tan et al., 2009; González et al., 2014) and geographies (e.g., Jones et al., 2016). However, taxonomic assignment of many of these isolates or sequence clones remains, in many cases, elusive, and the existence of potential cryptic species calls for a more exhaustive phylogenetic revision of the taxon.

Using a broader taxon sampling that spans the *Acidithiobacillus* species complex at a global scale, the 16S rRNA gene as marker and oligotyping as a strategy to differentiate closely related taxa (Eren et al., 2013), we have explored the evolutionary relationships of the different linages within the taxon and attempted to improve the phylogenetic resolution and better define the species boundaries within the sampled repertoire of *Acidithiobacillus* strains. Inter- and intraspecific levels of divergence were further examined using multilocus sequence analysis (MLSA). Also, occurrence and distribution of lineages and sequence variants in different acidic biotopes were explored by tracing oligotype profiles of sequenced metagenomes available in public databases.

## Methods

### DNA extraction, PCR amplification and sequencing

General culturing techniques used were as described previously (Acuña et al., 2013). DNA isolation and routine manipulations were carried out following standard protocols (Nieto et al., 2009). All amplicons were generated by PCR using the high fidelity polymerase (*Pfu* DNA Polymerase, Promega) and amplification parameters recommended by the manufacturer. Primer annealing temperatures for each reaction are indicated in Supplementary Table 1. PCR products were purified using the QIAquick PCR purification kit (Qiagen Inc., USA). Gene sequencing was performed by the Sanger method at Macrogen Inc. (Korea).

### Ribosomal operons and 16S rRNA gene sequences

Ribosomal RNA operon sequences of *Acidithiobacillus* type strains (T) and reference strains (R) were obtained from publicly available genomes deposited in Genbank (*A. ferrooxidans* ATCC 23270^T^ NC011761, *A. ferrivorans*^R^ DSM 22755 NC015942, *A. caldus* ATCC 51756^T^ CP005986 and *A. thiooxidans* ATCC 19377^T^ NZAFOH00000000. In the absence of a complete or draft genome sequence for the type strain (NO-37) of *A. ferrivorans* (DSM 17398), strain SS3 (DSM 22755) was used as a reference. The latter was previously confirmed as a strain of the (then) newly-described species, *A. ferrivorans* (Hallberg et al., 2010). Ribosomal RNA operon sequences for *A. ferridurans* ATCC 33020^T^, *A. ferriphilus* DSM 100412^T^ and *A. albertensis* DSM 14366^T^ were produced in house by PCR and subsequently sequenced. Accession numbers, annotations and coordinates of each operon are detailed in Supplementary Table 2. Downstream phylogenetic analysis was carried out using 529 16S rRNA gene sequences assigned to the genus *Acidithiobacillus*, available at GenBank as of July 2016, meeting length and quality requirements (Supplementary Table 3). Sequences for the 16S rRNA gene of 51 additional strains from our laboratory collection obtained by PCR were also included in the analysis (Supplementary Table 3). The PCR primers used are listed in Supplementary Table 1.

### 16S rRNA phylogenetic analysis

Small subunit ribosomal RNA gene sequences of 580 *Acidithiobacillus* strains and sequenced clones (Supplementary Table 3) were aligned using the MAFFT v7.229 software using the L-INS-I method (Katoh and Standley, 2013). The resulting alignments were trimmed and masked (>50%) manually. Phylogenetic trees were generated by two methods. First, a maximum likelihood tree was reconstructed using PhyML (Guindon and Gascuel, 2003), with the following settings: Tamura–Nei (Tamura and Nei, 1993) was used as substitution model, PhyML estimated the transition/transversion ratio and the proportion of invariant nucleotides and a discrete gamma approximation with *k* = 4. The topology of the tree and the length of the branches were optimized by PhyML, using Nearest Neighbor Interchange and Subtree Pruning and Regrafting. The phylogenetic tree was assessed using 1000 bootstrap replicates. A second tree was generated using Bayesian analysis with MrBayes v.3.0b4. Bayesian analysis was run for 3,000,000 generations, and trees were saved every 100 generations. Posterior probabilities were calculated after discarding the first 30% of trees (Huelsenbeck and Ronquist, 2001). Trees were visualized and annotated in FigTree (http://tree.bio.ed.ac.uk/software/figtree/).

### Oligotyping

Oligotyping of the 16S rRNA gene sequences was performed as described by Eren et al. (2013). A total of 12 positions with the highest entropy along the length of the sequence alignment (1054 nucleotides) were selected (Supplementary Figure 1), which spanned the following variable regions: V2 (46, 47, 62), V3 (257, 263, 264, 266), V4 (417, 418), V5 (588), and V6 (725, 765). Positions are defined with respect to the *Escherichia coli* 16S rRNA gene sequence. Oligotype (OT) assignment and sequence profiles for each individual strain or sequence clone, is indicated in Supplementary Table 3. Relative abundance, species-specific and lineage assignments of each of the major oligotypes (present in more than 3 individuals) and minor positional sequence variants (present in less than 3 individuals) scored from the raw data, are summarized in Supplementary Table 4.

### Multi-locus sequence analysis (MLSA)

MLSA markers were selected as described by Nuñez et al. (2014). Internal gene fragments for each marker were amplified by PCR, using primers listed in Supplementary Table 1 and genomic DNA obtained from 32 *Acidithiobacillus* strains from our laboratory collection, and sequenced. The same markers were derived from 13 publicly available genomes of *Acidithiobacillus* strains. Sequences were aligned with the MAFFT v7.229 software (Katoh and Standley, 2013) and manually curated, when appropriate. Concatenation of the MLSA markers was done with MEGA 6. The phylogenetic trees were constructed using maximum likelihood and Bayesian analysis. Bootstrap resampling was performed using 1000 replications to estimate the confidence of the tree topologies. Optimal models for nucleotide substitution, DNA polymorphism data, mean G+C contents, Tajimas D, and dN/dS ratios values, were all calculated using MEGA 6. Bayesian trees was constructed using MrBayes v.3.0b4 (3,000,000 generations, trees saved every 100 generations and posterior probabilities calculated after discarding the first 30% of trees (Huelsenbeck and Ronquist, 2001). Trees were visualized and annotated in FigTree (http://tree.bio.ed.ac.uk/software/figtree/). Sequence type assignments for each individual strain are indicated in Supplementary Table 5.

### PFGE with SpeI digestion and southern hybridization analysis

Whole cells (1 × 10^9^ cells/ml) were embedded in agarose blocks as described by Swaminathan et al. (2001). Genomic DNA was digested with 10 U/μl of the restriction endonuclease XbaI (ThermoFischer Scientific) at 37°C for 4 h. The resulting fragments were separated by Pulsed Field Gel Electrophoresis (PFGE) on a CHEF-DR III system (BioRad) in a 1% pulse field certified agarose gel (BioRad) at 6 V/cm and 14°C for 13 h with a pulse interval of 10 s. The gel was transferred by Southern blotting onto a Hybond-XL membrane (General Electric) and hybridized by incubation at 42°C for 1 h to a PCR-generated, biotin-labeled probe spanning a 294 bp internal fragment of the 16S rRNA gene (complementary to hypervariable region V4). The blot was developed with HRP-conjugated antibody and streptavidin to recognize the biotin-labeled probe. Chemiluminescent detection of horseradish peroxidase (HRP) enzyme activity was achieved with Pierce ECL western blotting substrate (Thermo Scientific).

### Statistical analyses

Various packages within the R software (version 3.1.12; http://www.r-project.org) were used for statistical analyses of the data and metadata. Principal component analysis (PCA) was used to evaluate the contribution of different quantitative parameters (as defined in Supplementary Table 3) to the variability of the data.

## Results

### rRNA operons in sequenced genomes of the acidithiobacilli

To assess the intra- and inter-specific diversity of *Acidithiobacillus* spp., the relationships of all known strains and sequence clones deposited in public databases (meta/genomic) were investigated using phylogenetic reconstruction strategies. Firstly, variations in copy number, operon architecture, and sequence were quantitated for each of the type (or reference) strains of the genus. This analysis showed that the number of rRNA operons is highly conserved within the genus (Table 1), with all sequenced strains having two copies of this operon. This was confirmed experimentally by Southern blot hybridization of *Xba*I digested and PFGE-fractionated genomic DNA (Supplementary Figure 2). Gene context of the operons I and II is distinctive (Supplementary Table 2) and is only partially conserved across the species reflecting lineage specific rearrangements. All operons surveyed have internal transcribed spacer (ITS-1) regions coding for tRNA^Ala^ and tRNA^Ile^ genes.

**Table 1:** **Intraspecific comparison of the rRNA operons I vs. II for type/reference strains within the *Acidithiobacillus* species complex**.

**Species**	**Operons**	**Intraspecies % sequence identity (Size in bp)**					Operon Context (^*^)
		**16S**	**ITS-1**	**23S**	**ITS-2**	**5S**	
*A. ferrooxidans*^T^ (ATCC 23270)	2	100	100	99.9	100	100	A–B
		(1546)	(428)	(2910)	(80)	(116)	C–D
*A. ferridurans*^T^ (ATCC 33020)	2	100	100	100	100	100	A–B
		(1536)	(454)	(2910)	(58)	(121)	C–D
*A. ferrivorans* (DSM 17398)	2	100	100	100	100	100	A–B
		(1538)	(441)	(2910)	(74)	(121)	C–D
*A. ferriphilus*^T^ (DSM 100412)	2	100	100	100	100	100	A–D
		(1526)	(454)	(2910)	(74)	(121)	C–B
*A. thiooxidans*^T^ (ATCC 19377)	2	100	99	99.9	100	100	A–D'
		(1536)	(458)	(2909)	(54)	(120)	C–B
*A. albertensis*^T^ (DSM 14366)	2	100	100	100	100	100	A–D' C–B
		(1536)	(458)	(2909)	(56)	(121)	
*A. caldus*^T^ (ATCC 51756)	2	100	100	100	100	100	A'–B
		(1540)	(380)	(2887)	(69)	(121)	C'–E

* *Operon context configuration details can be found in Supplementary Table 2*.

T *Type strain of the species*.

Intra-specifically, both operons were identical or nearly identical in size and sequence (Table 1). Inter-specifically, the operons analyzed varied in length by up to 130 bp (ranging from 4958 to 5088 bp), with variations correlating directly with variations in the size of ITS-1 regions (Table 1). Sequence differences among strains were mainly located within the ITS regions, between the tRNA^Ala^ and the 23S, and the 16S rRNA gene variable regions. At the level of the 16S rRNA gene, divergence between strains belonging to the currently recognized species showed considerable variation (Table 2). When all 16S rRNA gene sequences were compared, the reference strains showed an overall pairwise identity of 97.6%, with 1447 conserved and 91 variable sites. Most of the differences detected in nucleotide identity were located within the variable regions V3 and V4. Given the intrinsic information content of the 16S rRNA gene and the availability of sequences for this gene in public databases with respect to the ITS regions of the acidithiobacilli (1101 16S rRNA gene sequences versus 137 ITS sequences), only this marker was used in downstream phylogenetic analysis of the *Acidithiobacillus* species complex.

**Table 2:** **16S rRNA genes (upper half) vs. MLSA concatenate (lower half) identity matrix for *Acidithiobacillus* species complex type and reference strains**.

	**AFE**	**AFD**	**AFV**	**AFP**	**ATH**	**AAL**	**ACA**	
*A. ferrooxidans*^T^ (AFE)	–	**98.9**	98.3	98.5	97.8	98.0	96.1	16S rRNA Identity (%)
*A. ferridurans*^T^(AFD)	96.7	–	98.6	**99.2**	98.2	98.2	96.2	
*A. ferrivorans*^DSM17398^ (AFV)	87.4	87.2	–	**99.3**	97.8	97.8	96.0	
*A. ferriphilus*^T^(AFP)	88.0	87.6	96.1	–	97.8	97.8	96.3	
*A. thiooxidans*^T^(ATH)	80.4	80.6	77.4	77.5	–	**99.9**	95.8	
*A. albertensis*^T^(AAL)	80.5	80.5	78.0	79.2	96.0	–	95.8	
*A. caldus*^T^(ACA)	77.4	75.7	75.0	73.8	71.8	73.5	–	
	MLSA Concatenate Identity (%)							

*In bold, identity values above the accepted cutoff for species delineation (16SrRNA: > 98.7; MLSA > 97%)*.

T *Type strain of the species*.

Notably, when using the 16 rRNA gene as a marker to revise current species delineation (Table 2), a number of species pairs, namely *A. ferrooxidans-A. ferridurans, A. ferriphilus-A. ferridurans, A. ferriphilus-A. ferrivorans*, and *A. thiooxidans-A. albertensis*, exceeded the typical 97% and the conservative 98.7% sequence identity threshold values used as “gold standards” for species differentiation in the absence of DNA-DNA reassociation experiments (Stackebrandt and Goebel, 1994; Stackebrandt and Ebers, 2006).

### 16S rRNA gene-based phylogeny of *Acidithiobacillus* strains and isolates

To infer the phylogenetic relationships between all available strain and clone sequences of the acidithiobacilli, a comprehensive phylogenetic tree was constructed. For this, 16S rRNA genes were amplified and sequenced from a total of 53 representative strains belonging to the seven described *Acidithiobacillus* species and 1101 other gene sequences belonging to isolates and uncultured clones, retrieved from GenBank. After filtering for redundancy and sequence length, applying masks to positions with >50% gaps and eliminating ambiguous characters, a final set of 580 sequences was obtained (Supplementary Table 3). This set encompassed 1054 bp of the full 16S rRNA gene sequence and contained 642 variable sites and 275 parsimony informative sites, (97.3% pairwise identity). Within this data set, 74.5% of the sequences had taxonomic assignment to the species level. *Thermithiobacillus tepidarius*, the type species of the single other family within the order *Acidithiobacillales*, was included as the outgroup.

**Figure 1:**
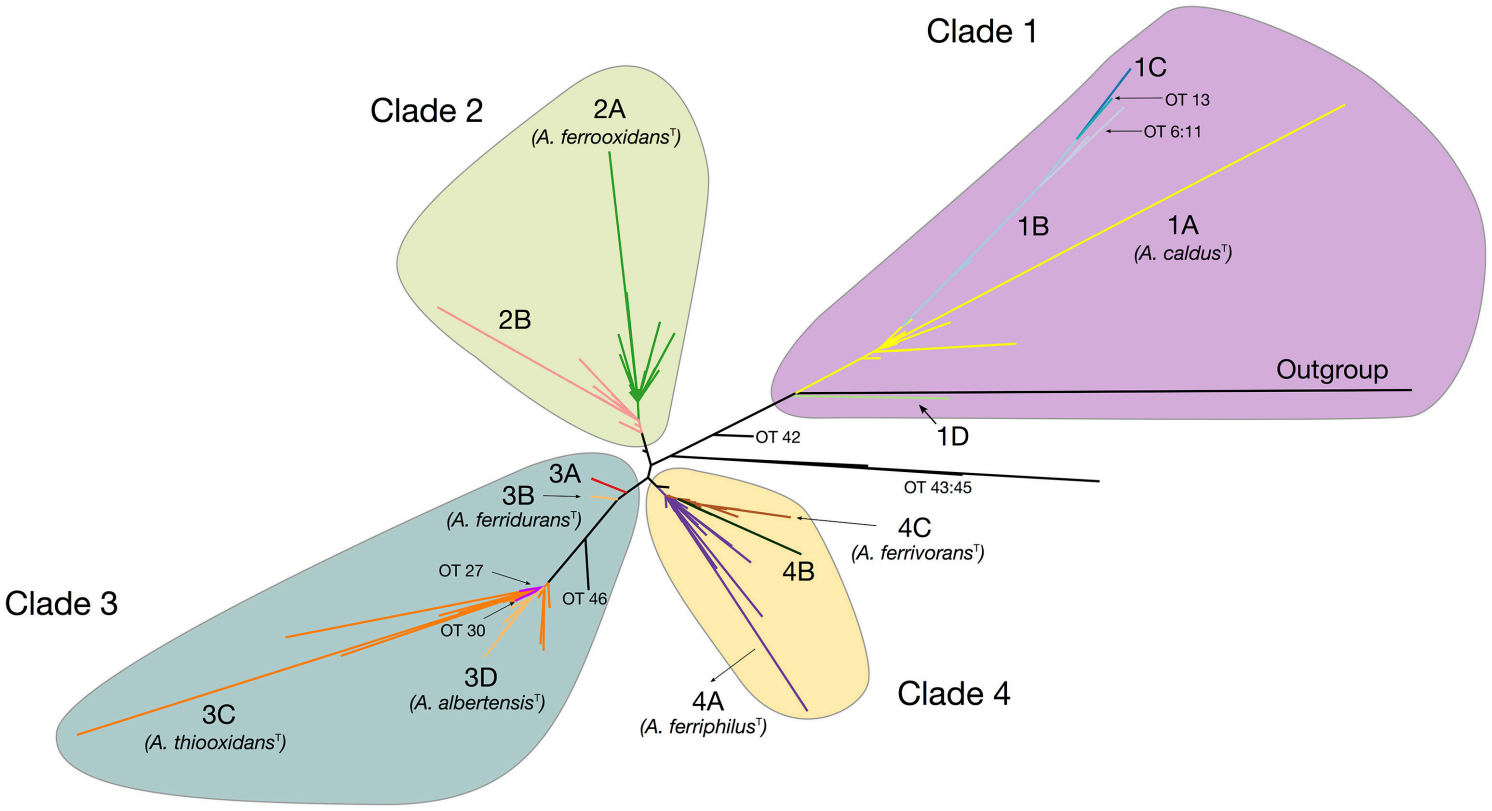
***Acidithiobacillus*** **species complex consensus phylogenetic tree built using maximum likelihood inference and 16S rRNA gene sequences of 580 strains and/or sequence clones**. Clade's affiliations are as follows: clade 1 (purple, *A. caldus*^T^), clade 2 (green, *A. ferrooxidans*^T^), clade 3 (turquoise, *A. ferridurans*^T^, *A. thiooxidans*^T^, *A. albertensis*^T^), clade 4 (yellow, *A. ferriphilus*^T^, *A. ferrivorans*^T^). Subclades 1A through 4C are color coded according to the oligotype (OT) assigned to the strains and sequence clones that conform each cluster. Minor branches of interest are also shown in the tree and the corresponding OTs indicated. Detailed information on the sequences used in the tree construction and clade assignments can be found in Supplementary Table 3.

The maximum likelihood (ML) phylogenetic tree built for this dataset is shown in Figure 1. An additional tree built using Bayesian inference can be found in the Supplementary Material (Supplementary Figure 3). The ML tree obtained shows a clear separation of all sequenced representatives in 4 distinctive clades including the *A. caldus* strains (Clade 1; ATCC51756^T^), the *A. ferrooxidans* strains (Clade 2; ATCC 23270^T^), the *A. ferridurans-A. thiooxidans-A. albertensis* strains (Clade 3; ATCC 33020^T^, ATCC 19377^T^, DSM14366^T^) and the *A. ferrivorans-A. ferriphilus* strains (Clade 4; DSM 22755, DSM 100412^T^). Support values for the principal nodes were generally high (>70%). There was limited disagreement in topology between trees built with ML and Bayesian inference (Supplementary Figure 3), with discordance restricted to placement of the *A. ferriphilus-A. ferrivorans* clade and a few other less strongly supported nodes. Also, sister-clades with diverse node depth became apparent in all four branches of the tree (e.g., subclade within the *A. caldus* Clade 1). These are further analyzed below.

### Sequence entropy-based analysis of the phylogenetic structure of the genus

Oligotyping (Eren et al., 2013) was used to improve resolution of the 16S rRNA gene tree of the *Acidithiobacillus* strains. This approach utilizes a sequence entropy-based method to identify the most informative nucleotide positions within a surveyed set of sequences. This has proven useful in subspecies-level analysis and is of value for detecting ecologically distinct organisms within closely related taxa (Eren et al., 2015). Using the depurated set of sequences that entered into the phylogenetic analysis, we explored whether there were strong correlations between the clades in the consensus tree representation and their original taxonomic assignments and the oligotypes derived for each sequence (Figure 1). For this purpose, the entropy at each nucleotide position in the sequence alignment was calculated, resulting in 12 information-rich positions spanning variable regions of the 16S rRNA gene V2–V9 (Supplementary Figure 1).

A total of 46 oligotypes (OTs) were derived from the dataset (Supplementary Table 4). Of these, 28 OTs were present in a single or less than 3 sequences and were omitted from further analyses. Poor representation in the dataset of the positional variants of other more abundant OTs could be indicative of randomly-generated diversity emerging from sequencing errors, especially if homogenously distributed around the tree. Interestingly, sequence variability was higher in certain clades and in those clades the positional variants map to larger branches of the tree and or to the tips of the tree. This indicates that a number of these OTs are descendants of recent origin and imply recent diversification of the dominant OT (e.g., OTs 6–13 in Clade 1). A number of the single-sequence OTs mapped to deep branches of the tree (OTs 42–46), and could possibly represent ancestral strains or still cryptic phylotypes.

All of the clades in the tree depicted in Figure 1 were found to group 2–4 principal OTs (defined as having more than 3 representative sequences). Seven of these OT-defined groups (OTs 1, 15, 23, 24, 28, 31, 39) exactly match the tree branches defining currently recognized *Acidithiobacillus* species (Table 3, Supplementary Table 4). However, additional OTs matching unassigned (or mis-assigned) sequence representatives clustering in coherent subclades or sister clades within the tree were also identified (Figure 1, Table 3). This is the case of subclade 2B in the *A. ferrooxidans* branch (represented by strain DSM 1927), which accommodates sequences representing 41 strains and/or sequence clones baring a distinctively different oligotype (OT18). Sequences displaying oligotype OT6 (12 in total), OT7 (5 in total), OT12 (9 in total), and OT13 (3 in total), cluster in a shallow branch within the *A. caldus* clade, despite of the fact that a number of these had been deposited as sequence representatives of *A. thiooxidans*. These sequences are highly divergent from the 16S rRNA gene sequence of the type strain of *A. caldus* (95–96.5% % identity) and seem to comprise different (sub)species. A similar scenario applies to a number of other smaller branches within the *A. thiooxidans*-*A. albertensis* clade (Clade 3, OT27 and OT30) and the *A. ferriphilus*-*A. ferrivorans* clade (Clade 4, subclade 4B) that correlate with specific oligotypes (Table 3). In addition, based on this approach, most of the unassigned sequence representatives deposited in public databases can now be assigned to a species or candidate phylotype (Table 3, Supplementary Table 3).

**Table 3:** **Oligotype distribution with respect to original taxonomic assignments and subclades in the consensus tree representation**.

**Subclade**	**Oligotype**		***A. caldus***	***A. ferrooxidans***	***A. ferridurans***	***A. thiooxidans***	***A. albertensis***	***A. ferriphilus***	***A. ferrivorans***	***Acidithiobacillus*** **spp**.	**Total**	**Rep. strains**
1A	1	AGCCCGTCGTCG	**30**	0	0	0	0	0	0	19	49	ATCC 51756^T^
2A	15	GACTCATTTACG	0	**99**	0	0	0	0	0	22	121	ATCC 23270^T^
3B	23	GGTTCATCGCCG	0	27	**8**	0	0	0	0	3	38	ATCC 33020^T^
3C	24	AATGTCCTTATA	0	1	0	**71**	0	0	0	15	87	ATCC19377^T^
3D	28	AATGACCTTATA	0	2	0	14	**10**	0	0	12	38	DSM 14366^T^
4A	31	GGTCCGTCCACG	0	76	0	0	0	**8**	1	10	95	DSM 100412^T^
4C	39	GATCCGGCAACG	0	4	0	0	0	0	**12**	5	21	DSM 22755^T^
1B	6	AGCGTCTCGTAT	1	0	0	0	0	0	0	11	12	LA10A
1B	7	GGCGTCTCGTAT	0	0	0	0	0	0	0	5	5	NJU-AMD3
1C	12	TGCGTCGCGTGC	0	0	0	4	0	0	0	5	9	ZMB
1C	13	AGCCTGTCGTGC	1	0	0	0	0	0	0	2	3	ORCS6
1D	14	GACCCGTTGACG	0	0	0	0	0	0	0	3	3	NJU-T1
2B	18	GACTCATCCACG	0	37	0	0	0	0	0	4	41	DSM 1927
3A	22	GGTCCGTCCCCG	0	1	0	0	0	0	0	4	5	LMT1
3D	27	AATGTCCTTCTA	0	0	0	8	0	0	0	1	9	GG1/14
3D	30	AATGCCCTTATA	0	0	0	4	0	0	0	0	4	ATCC 21835
4B	37	GGTCCGGCCACG	0	0	0	0	0	0	0	7	7	BER_D10
4C	40	GATCCGGCCACG	0	0	0	0	0	0	0	3	3	NJUST22
Rest		NNNNNNNNNNNN	0	10	0	2	0	0	0	18	30	All others
**Correctly assigned**^a^			30	99	8	71	10	8	12	−		
**Unassigned**^b^			19	22	3	15	12	10	5	149		
**Mis-assigned**^c^			2	158	27	32	16	77	4	31		

T Type strain of the species

a *Number of strains assigned to the taxon that possess the oligotype of the type strain of the species (bold)*.

b *Number of strains without a specific assignment that possess the oligotype of the type strain of the species*.

c *Number of strains assigned to the taxon that possess the oligotype of the type strain of a different species*.

### Oligotypes occurrence and prevalence in acidic econiches around the globe

Following this, we explored whether the diversity uncovered represents the global origins of isolates and clones, or whether it could be explained by niche-specific selective pressures or associated to specific environmental cues. For this, we collected all available metadata published in the literature or deposited in public databases for the strains included in the analysis (Supplementary Table 3) and performed basic statistical analyses. Also, occurrence and relative abundance of the different subclades in publically available targeted metagenomic datasets were scored (Supplementary Table 6), and the derived information was analyzed in the context of strain-specific data.

**Figure 2:**
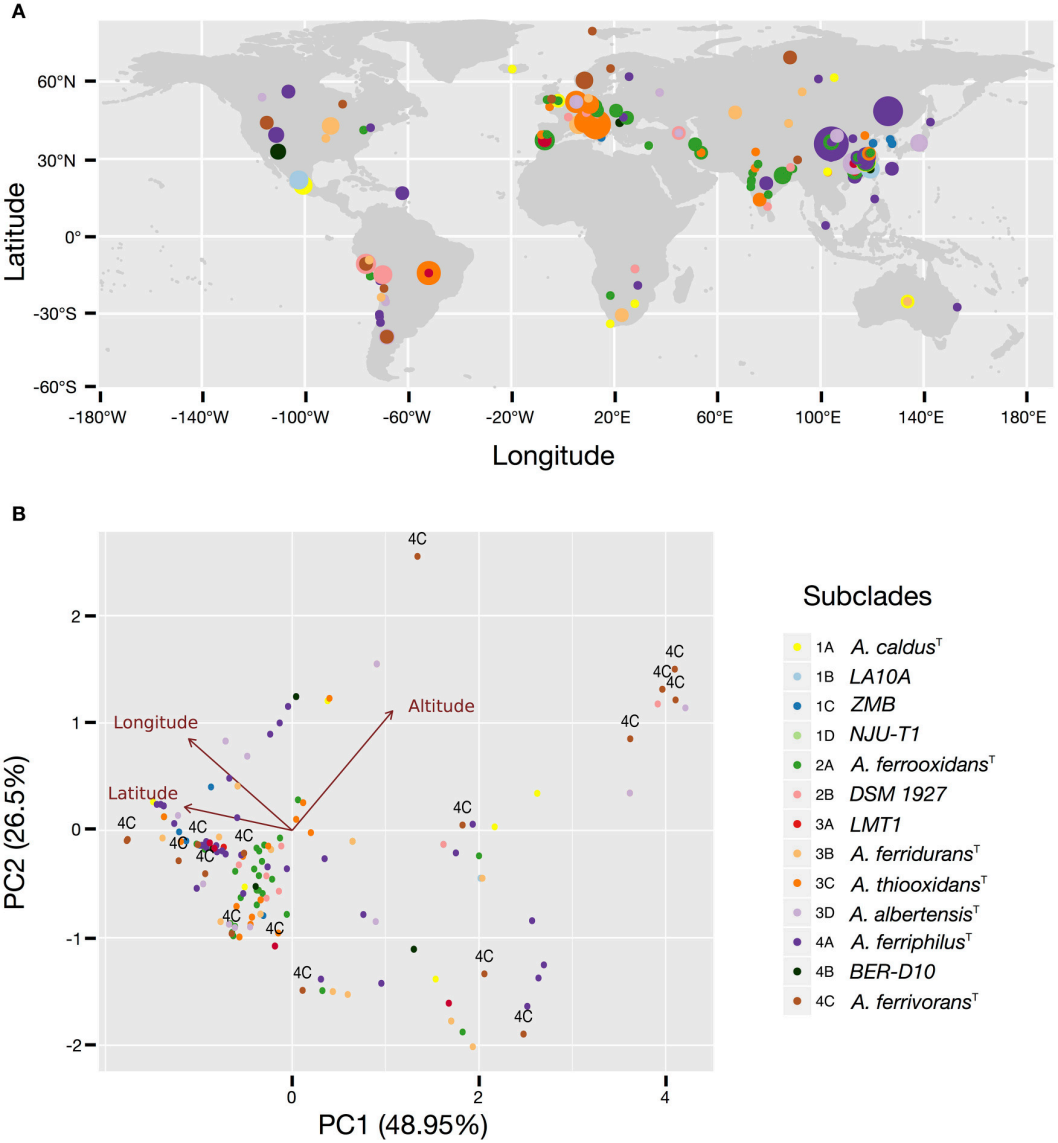
**Occurrence and prevalence of the *Acidithiobacillus* species complex subclades in acidic econiches around the globe**. **(A)** Location and relative abundance of each subclade according to latitude and longitude coordinates. **(B)** Principal component analysis (PCA) biplot showing arrangement of the isolates and sequence clones according to variations in latitude, longitude and elevation. Individuals are color coded according to the 16S rRNA gene tree subclade they belong to. Taxon assignment of the different subclades is indicated in the figure, by the species name or the name of a representative strain. Arrows represent the relationship (direction and strength) of the parameters with the individuals. The direction of each arrow indicates an increase in that variable.

More than 50% of all *Acidithiobacillus* strains and sequence clones sampled can be mapped to Asiatic countries (45% of which originated in China), followed by Europe (20%), South (9%), and North America (7%), and Africa and Oceania (accounting for less than 2% each). Similarly, more than 60% of all strains and sequence clones have been obtained from industrial econiches. Both figures imply that some of the tendencies emerging from the data may be obscured by sampling biases. Despite this fact, it is clear that several subclades are ubiquitous worldwide (Figure 2), including the subclades represented by the type strains of *A. caldus* (subclade 1A), *A. ferrooxidans* (subclade 2A), *A. ferridurans* (subclade 3B), and *A. ferriphilus* (subclade 4A). However, differential patterns of occurrence and/or relative abundance are also evident from the map in Figure 2A and are generally consistent between natural and industrial econiches (Supplementary Figure 4). The strongest apparent tendency is the decrease in the diversity of subclades detectable at increasing latitudes (Figures 2A,B), which is also evident from the subclade assignments derived from targeted metagenomic data (Supplementary Table 6). Prevalence of the *A. ferriphilus* (4A) subclade in China, the *A. ferrooxidans* (2A) subclade in European countries and India, the *A. thiooxidans* (3C) subclade in Europe and Brazil and the *A. ferrooxidans*–like (2B) subclade in South and North America is notably high. In contrast, subclade 4C represented by *A. ferrivorans* SS3 and related strains, is restricted to high latitudes and high altitudes (Figure 2B, Supplementary Table 3), being the only *Acidithiobacillus* type found at the most extreme latitudes. This is in agreement with the psychro-tolerance of known strains from the clade (Hallberg et al., 2010). Further support for this finding emerges from targeted metagenomic data obtained at the coal mine in Svalbard, Norway, and a copper tailing in Ontario, Canada, were every representative of the genus *Acidithiobacillus* detected matches the 16S rRNA gene oligotype of clade 4C, represented by *A. ferrivorans* SS3.

Evidence for niche-specific diversification of certain clades is also apparent from the available data, as in the case of Clade 1, grouping *A. caldus*-like strains. While subclade 1A, represented by the *A. caldus* type strain, is present in different global locations and mostly associated with industrial operations involved in copper and gold recovery or in coal exploitation, subclades 1B and 1C are restricted to copper sulfide mining sites in China and subclade 1D to zinc/lead ores mined in USA (Supplementary Table 3), suggesting that local selection pressures (eventually process-specific) are driving differentiation of ecotypes. Occurrence of the 1D subclade in the targeted metagenomes obtained from the Iron King Mine tailings in Arizona (USA) and dominance of clade 1D over other *Acidithiobacillus* species in the metagenomes obtained from the Chinese mine tailings in the Tongling region, both of which are heavily polluted with metalloids (Huang et al., 2012; Hayes et al., 2014), could provide further hints on the drivers behind the diversification of the *A. caldus* linage. The same argument stands for the 1B subclade, which is highly abundant in sulfidic caves from Mexico and also dominate the *Acidithiobacillus* population in concrete pipes in Ala Moana Park (Hawaii), as assessed by targeted metagenomics (Supplementary Figure 4; Supplementary Table 3). In agreement with this finding, strains originating in Mexico were recently suggested to represent a new species (Jones et al., 2016).

### MLSA-marker based phylogeny of sequences from strains and isolates

MLSA was used to gain deeper insight into the genetic structure of the *Acidithiobacillus* species complex at a higher resolution level. Informative markers were selected using a previously developed scheme for identification of housekeeping genes suitable for MLSA (Nuñez et al., 2014). All 10 genomic sequences of validated *Acidithiobacillus* spp. available in public databases as of July 2016 were used as input in this analysis (Valdés et al., 2008, 2009, 2011; Liljeqvist et al., 2011; You et al., 2011; Talla et al., 2014; Travisany et al., 2014; Yin et al., 2014; Yan et al., 2015; Latorre et al., 2016). Eight HKG that met the amplicon size requirements of the pipeline were selected for further phylogenetic analysis. Internal gene sequences of the 8 markers were amplified from genomic DNA obtained from an additional set of 35 *Acidithiobacillus* strains and industrial isolates of diverse geographical origins by PCR, using a high fidelity polymerase. Details on the marker genes, the allelic profiles, and the sequence types (ST) derived from sequence analyses, are summarized in Table 4, and the GenBank accession numbers for the sequences generated in this study are listed in Supplementary Table 5. The concatenate comprised 4086 nucleotides and consisted of 1832 variable sites. The eight protein-coding gene loci showed a mean nucleotide sequence diversity of 34.2%, in contrast with that using the 16S rRNA gene alone, which yielded only 5.4% polymorphic sites. Parsimony informative sites, i.e., positions in the sequence set under comparison that contain at least two types of nucleotides in at least two different sequences, varied form a maximum of 368 (*ruvB*) to a minimum of 79 (*ihfB*).

**Table 4:** **Sequence analysis of the MLSA selected markers and the 16S rRNA gene**.

**Locus**	**Length (bp)**	**N° Alleles**	**Parsimony informative sites**		**Mean G+C**	**Tajima's D**	**dN/dS**
			**N°**	**%**			
*rrs*	1216		66	5.4	56.7	1.080	–
*IlvE*	890	22	322	36.2	57.9	0.724	0.095
*trx?*	296	20	108	36.5	50.7	1.006	0.060
*rpsE*	485	26	190	39.2	56.6	0.808	0.585
*ruvB*	848	27	324	38.2	62.3	0.647	0.522
*rplU*	290	22	104	35.9	57.9	1.051	0.141
*ihfb*	225	22	81	36.0	54.2	1.428	0.066
*hsIV*	434	24	153	35.3	61.5	0.571	0.089
*fdx*	281	22	111	39.5	58.0	1.140	0.185

**Figure 3:**
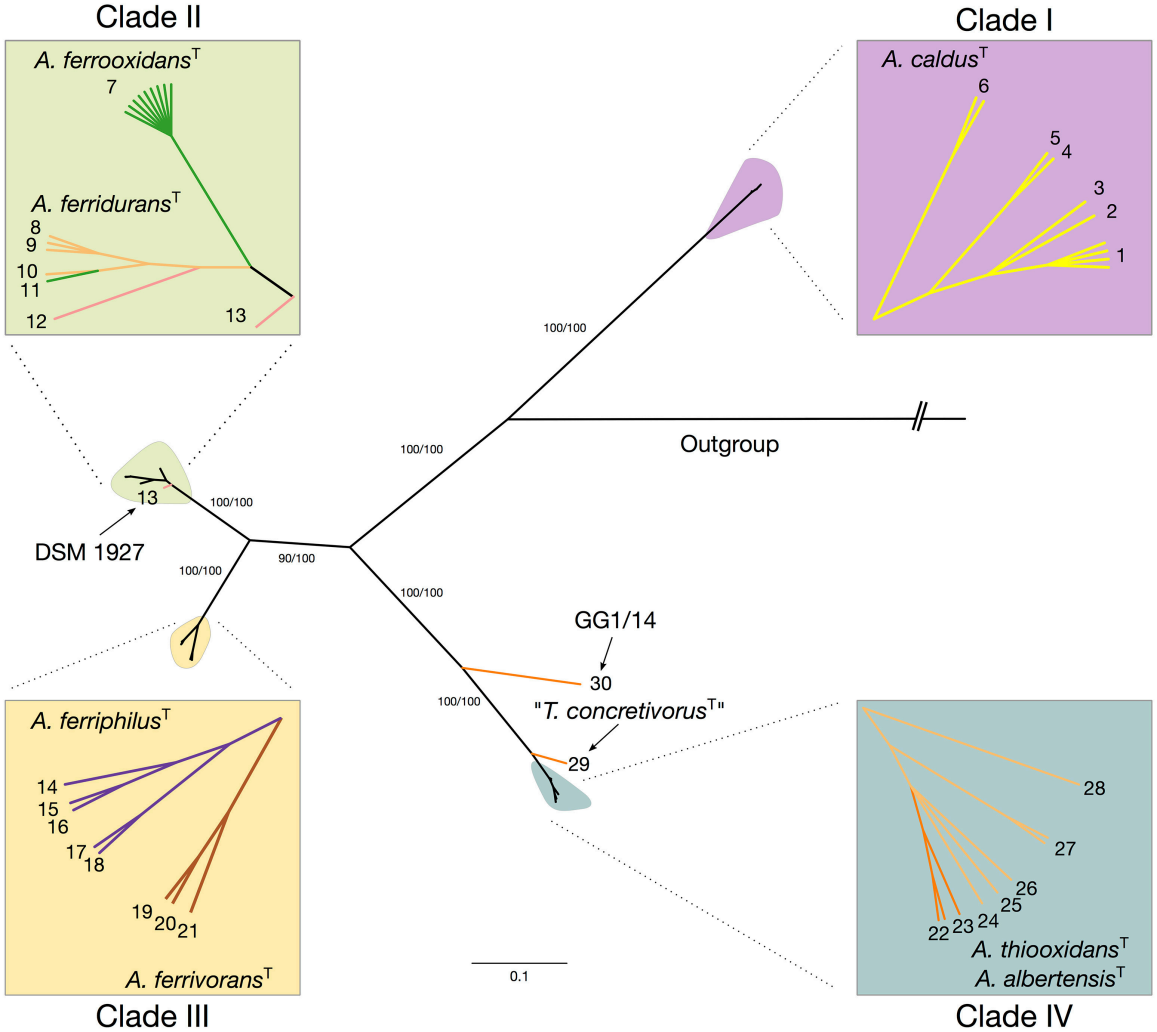
**Maximum likelihood tree inferred from the concatenate of eight selected MLSA markers**. The *ilvE, trx, rpsE, ruvB, rplU, ihfB, hsIV*, and *fdx* gene sequences (4086 bp) from 45 *Acidithiobacillus* strains representative of available subclades undercovered in the 16S rRNA analysis were concatenated and analyzed using MEGA 6. Numbers at the nodes indicate the bootstrap values of 1000 replicates (%). The bar represents expected nucleotide substitutions per site.

Maximum likelihood and Bayesian inference based phylogenetic trees were constructed using a sequence concatenate of all eight markers. Topology of the concatenate-based tree was congruent between methods and with the topology of single-gene trees generated with most informative markers, suggesting that none of the markers utilized was the object of active gene flow (Supplementary Figure 5). Phylogenetic analysis of the concatenate produced 6 major clades supported by bootstrap values of > 93% (Figure 3). Despite some disagreement in the topology of the MLSA-based tree and the 16S rRNA gene-based tree, mostly due to difference in the divergence times between sister clades, all clades identified coincided with the major clades emerging from the 16S rRNA gene phylogenetic analysis (see Figure 1). Discordance occurred in the placement of the *A. ferridurans* subclade in both trees.

Based on the concatenate alignment, the 45 isolates were resolved into 30 STs (Supplementary Table 5). All clades showed high variability in terms of STs, which may be explained by the non-clonal nature of the strains analyzed, many originated from different sources and environments (Supplementary Table 3). All eight protein-coding loci that comprised the concatenate have nucleotide substitution ratios (dN/dS) well below 1, indicating pressure to conserve the gene sequences (Table 4). Overall, these values indicate that most of the sequence variability identified can be explained by strong negative selection, typical of housekeeping genes. However, inspection of the dN/dS ratios within specific branches of the trees generated with single genes showed positive values in some clades and subclades. Specifically, 7 out of 8 trees showed dN/dS ratios above 1, indicating positive selection for the selected genes analyzed within clade IV. The same holds true for clade III in 3 out of 8 trees. These differences between general ratios and ratios observed at individual branches of the tree, may be explained by the lesser time since divergence of the subclades conforming each of these two clades. MLSA distances between subclades within each clade are near the threshold for species delineation, hinting that events of speciation are still ongoing, supporting the observation of high levels of adaptive evolution on the analyzed markers.

### Levels of diversity within and across lineages

**Figure 4:**
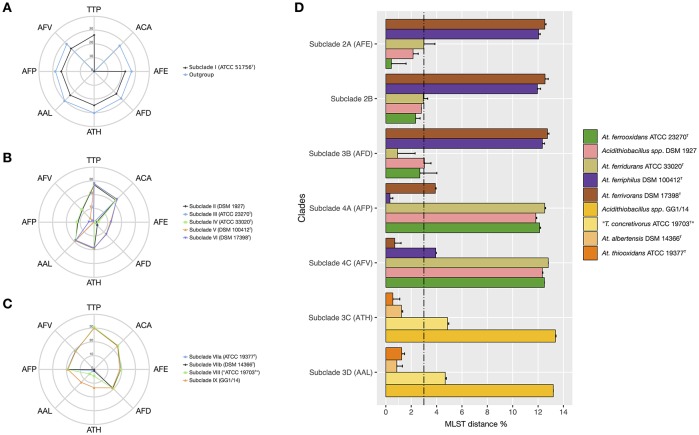
**Average nucleotide divergence of the MLSA concatenate (%) between strains conforming each of the MLSA clades against all reference species in the *Acidithiobacillus* species complex and the outgroup *Thermithiobacillus tepidarius*. (A)** *T. tepidarius* and Clade I strains: (10 *A. caldus* strains); **(B)** Clades II and III strains: *A*. *ferrooxidans* (9 strains), *A. ferridurans* (7), *A. ferriphilus* (5 strains), *A. ferrivorans* (3 strains), and the variant lineage represented by strain DSM 1927; **(C)** Clade IV strains: *A*. *thiooxidans* (3 strains), *A. albertensis* (6 strains), and variant lineages represented by strains ATCC 19703 ex. *T. concretivorus*^T^ and GG1/14; **(D)** Average nucleotide divergence of the MLSA concatenate (%) within and between *Acidithiobacillus* species complex 16S rRNA subclades 2A-2B, 3A-3D; 4A, 4C. For each bar, the standard deviation is indicated. The 3% divergence threshold value is indicated (Vandamme and Peeters, 2014). Abbreviations: *Thermithiobacillus tepidarius* (TTP), *A. caldus* (ACA), *A. thiooxidans* (ATH), *A. albertensis* (AAL), *A. ferrooxidans* (AFE), *A. ferridurans* (AFD), *A. ferriphilus* (AFP), *A. ferrivorans* (AFV).

To further assess the levels of diversity within and across lineages at a higher resolution level, pairwise distances between a set of 45 *Acidithiobacillus* strains, including the reference strains of all seven validated species of the genus (Table 2; Figure 4), were calculated using the MLSA concatenate as molecular marker. As shown in Table 2, all seven *Acidithiobacillus* species are supported by MLSA concatenate divergence values larger that the 3% threshold value (meeting the 70% DNA-DNA hybridization threshold) used to differentiate strains into species in other microbial groups (Vandamme and Peeters, 2014).

The intergroup average divergence levels varied significantly depending on the lineage considered (Figure 4). Average divergence between *A. caldus* strains and the rest of the *Acidithiobacillus* species complex was almost as high as that observed for the related genus *Thermithiobacillus tepidarius* ATCC 43215^T^ (Figure 4A), raising the question if intrinsic differences are larger than those expected for species of the same genus. In turn, *A. ferrooxidans* was strongly differentiated from both *A. ferriphilus* (12.1 ± 0.1% divergence) and *A. ferrivorans* (12.5 ± 0.0% divergence), but only 3.0% divergent from *A. ferridurans*.

When additional strains were considered, the average within-group divergence for most lineages emerging from the 16S rRNA gene and/or the MLSA phylogenetic analyses (Figure 4) remained below the 3% threshold, with one exception. A considerable level of intragroup diversity distributed in 3 recognizable subclades was apparent within the *A. thiooxidans* clade (Figure 3). One subclade spanned the acknowledged species *A. thiooxidans* and *A. albertensis*, which were less than 3% divergent between each other. The other two subclades encompassed two potentially new species represented by strains ATCC 19703 (“*Thiobacillus concretivorus”*) and strain GG1-14, respectively. For these two novel taxons the average sequence divergence of the concatenate toward its nearest neighbor, *A. thiooxidans*^T^, was 4.9 ± 0.1% and 13.35 ± 0.05%, respectively. These figures supported their assignment as distinct *Acidithiobacillus* species. On the other hand, the *A. ferrooxidans*-like subclade 2B represented by strain DSM 1927, emerging from the combined 16S rRNA gene phylogenetic analysis and the oligotyping analysis, had an intertaxon divergence of 2.8% from the *A. ferrooxidans* subclade 2A (Figures 4C,D) which did not support recognition of separate species, even though levels of divergence at the MLSA concatenate level and the 16S rRNA gene levels indicated ongoing differentiation.

## Discussion

The class *Acidithiobacillia* currently consists of 9 validly described species arranged within a single order, the *Acidithiobacillales*. This order contains two genera: *Thermithiobacillus*, with two validated species [*T. tepidarius* (Hudson et al., 2014) and *T. plumbiphilus* (Watanabe et al., 2016) and *Acidithiobacillus* (Kelly and Wood, 2000)], which currently accommodates seven validated species. Despite the extensive work that has been carried out on the acidithiobacilli, the evolutionary relationships amongst members of this group remain poorly understood. In this study, we have generated the most thoroughly sampled species-level phylogeny to date for this group, using 16S rRNA gene sequence data and a MLSA scheme based on 8 single-copy orthologous genes represented across members of the *Acidithiobacillales*. Our aim was to produce a robust phylogeny by enabling rooting of the trees and to obtain a better understanding of the evolution of the taxon. In order to span both inter- and intra-species variation we assembled a diverse set of strains and sequence clones (>580) originating from sites throughout the world and encompassing all currently recognized *Acidithiobacillus* species, as well as neglected candidate species (e.g., “*T. concretivorus”*). The phylogeny resulting from the 16S rRNA gene data achieved a high coverage of the diversity of the group, while the oligotyping and the MLSA typing approaches enabled a more thorough inspection of the intra-clade diversity for a set of well sampled clades.

Based both on 16S rRNA gene- and the MLSA-based phylogenies, the majority of the sampled diversity clustered together with known reference strains and formed well-defined clades, supporting the seven (species) fundamental units previously described. However, a number of additional lineages (phylotypes) with statistically well-supported nodes also became apparent in either, or in both, analyses, uncovering further inherent diversity for this taxon. This is in agreement with evidence derived from phylogenetic studies encompassing more restricted sets of strains, mostly focused on iron-oxidizing strains of the group (Amouric et al., 2011; Wu et al., 2014 and references therein), which have resulted in recent reclassification of a number of strains of *A. ferrooxidans* into the new species referred to above. In this work a total of 6 new phylotypes (16S rRNA clades 1B, 1C, 1D, 2B, 3A, 4B, Supplementary Table 4) were identified that are readily distinguishable by phylogenetic clustering and important levels of sequence divergence. Taken as a whole, the evidence suggests that the *Acidithiobacillus* genus is better defined as a species complex made up of at least 13 phylotypes in diverse stages of differentiation (speciation) that can be distinguished from each other on the basis of divergent phenotypic and/or genotypic characters. Nonetheless, due to the continuous nature of evolution and in some cases the under-sampling of isolates or the scarcity of sequence representatives of certain phylotypes available, several of these phylotypes have remained cryptic and have not yet been adequately framed as discrete units or species.

Recognition of these new lineages has been further obscured by the extensive taxonomic mis-assignment of strains to named species, which has hidden a great deal of the inherent diversity of the *Acidithiobacilliae*. For many years, knowledge on the taxonomic structure of the *Acidithiobacillus* species complex has relied on classifications achieved on the basis of morphological and physiological characteristics. Acidophilic rods catalyzing the dissimilatory oxidation of both iron and sulfur have almost always been classified as strains of *A. ferrooxidans*, while those that only oxidize sulfur have been assigned to *A. thiooxidans* or *A. caldus*, depending on the optimal temperature of growth of the isolate. According to our 16S rRNA gene oligotyping results, 35.6% of the isolates, which presumably have been experimentally diagnosed before being assigned to a particular taxon, were actually incorrectly classified (e.g., *A. caldus* 1B as *A. thiooxidans*). This indicates that in many cases strains are not thoroughly evaluated in terms of their phenotypic features, and are somewhat arbitrarily assigned to one of these taxons without a systematic assessment of diagnostic characteristics (such as their optimal temperature ranges). Even in the case of sequence clones, where 16S rRNA gene data are the only piece of information available on the individual being sampled, major mistakes in the specific assignment were detected (17.6% of mis-assignments). A certain degree of mis-assignment is expected for data originating before the revision of the taxon, yet many of the mistakes correlate to recent data. In addition, a large number of sequences deposited in public databases (~40%) remain unclassified, while our data strongly support their specific assignments.

According to polyphasic taxonomy, strains of the same species should have similar phenotypes, genotypes, and chemotaxonomic features (Gillis et al., 2005). Currently, genotypic criteria required to differentiate species require strains to have <70% DNA-DNA hybridization similarity, > 5°C Δ T_m_, > 5% mol G + C difference of total genomic DNA, and a 16S rRNA gene divergence larger than 1.3% (Stackebrandt and Ebers, 2006). Measurement of all these parameters is seldom achieved for any particular strain, unless phenotypic, molecular and/or available genomic data support a specific reassignment, and confirmation is deemed necessary. From the genotypic standpoint, currently recognized *Acidithiobacillus* species have been evaluated only in some of these aspects, and in those actually tested thresholds values are not always met, making species delineation further unclear. DNA–DNA hybridization between *A. ferrooxidans* and either *A. thiooxidans* (9%, Harrison, 1982), *A. ferrivorans* (37%, Hallberg et al., 2010), or *A. ferridurans* (63%, Amouric et al., 2011) meet the 70% gold standard that serves as a boundary to differentiate species. Conversely, mean differences in total G + C content between each of the newly designated iron-oxidizing species and *A. ferrooxidans sensu stricto*, are in all cases lower than the 5% required to differentiate species. Mean differences in this parameter for the non iron-oxidizing species *A. thiooxidans, A. caldus* and *A. albertensis* with respect to *A. ferrooxidans* (6.8, 5.1, and 2.7 % respectively) have also a certain degree of ambiguity. These differences could result from varying degrees of horizontal gene transfer (HGT), blurring the boundaries at the level of the core genome between bacterial groups. In recent years, evidence has accumulated for the extensive contribution of HGT to the genomic evolution of the *Acidithiobacillus* species complex (e.g., Bustamante et al., 2012; Acuña et al., 2013; Travisany et al., 2014).

Our 16S rRNA gene sequence data support the differentiation of *A. ferrooxidans, A. ferrivorans, A. thiooxidans*, and *A. caldus*, all of which are more than 1.3% divergent with respect to all other currently recognized species in the complex, though this parameter failed to differentiate *A. ferrivorans* from *A. ferridurans, A. ferriphilus* from both *A. ferridurans* and *A. ferrivorans*, and *A. albertensis* from *A. thiooxidans*. Despite this fact, all seven acknowledged species in the complex are individualized as neat clades in the 16S rRNA tree and identified by a principal/predominant oligotype, supporting actual differentiation of these phylotypes. Overall the topology of our 16S rRNA-based tree is similar to those reported previously (e.g., Ni et al., 2008), with differences attributable mostly to the number of sequences, the wider diversity of sequences considered in the analysis and rooting using *Thermithiobacillus tepidarius* as outgroup. One interesting observation emerging from this deep coverage and rooted 16S rRNA tree is the relationship between species that can and cannot oxidize sulfur. Historically, *A. caldus* and *A. thiooxidans* have been considered to be more closely related to each other than to *A. ferrooxidans* (Goebel and Stackebrandt, 1994). However, the 16S rRNA gene phylogeny constructed herein placed *A. caldus* far from the rest of the acidithiobacilli and actually much closer to the outgroup species (*T. tepidarius*). In turn, based on this marker all *A. thiooxidans* related strains appear to have shared a more recent common ancestor with the iron-oxidizing lineages.

To achieve a more precise delineation of relevant operational units, microbial taxonomists are rapidly turning to genome-wide molecular markers, such as MLSA genes, genomic signatures, or even full sequences (Vandamme and Peeters, 2014). Genome sequences for number of *Acidithiobacillus* spp. have become available in public databases since 2008, including the type strains of *A. ferrooxidans, A. caldus, A. thiooxidans*, and *A. ferrivorans* strain SS3 (Valdés et al., 2008, 2009, 2011; Liljeqvist et al., 2011; You et al., 2011; Talla et al., 2014; Travisany et al., 2014; Yin et al., 2014; Yan et al., 2015; Latorre et al., 2016; Zhang et al., 2016a). However, no genome, complete or draft, has yet been reported for *A. ferridurans, A. ferriphilus* and *A. albertensis*. This poor representation of the acidithiobacilli in genomic databases hampers the detailed investigation of the evolutionary relationships of its members through thorough phylogenomic analyses (although efforts in this direction have recently been published; Zhang et al., 2016a,b), and prevents the unraveling of the ambiguities addressed above without further genome sequencing. Therefore, to achieve a high degree of resolution and at the same time restrict our view on the evolution of the taxon to its core genome (preventing biases introduced by HGT and recombination) we used single copy orthologous genes shared by all members of the complex as MLSA markers for further phylogenetic reconstruction. In the absence of robust genomic data, core genes-based MLSA has proven useful in understanding the evolutionary relationships and classification of other complex taxonomic groups (Konstantinidis et al., 2006).

Using MLSA analysis, the type/reference strains of all validated species clustered to discrete branches of the tree, regardless of the tree construction method used. However, at this level of resolution inclusion of some lineages as species of the genus appears unrealistic (*A. caldus*), and individualization of others (*A. thiooxidans-A. albertensis*), questionable. MLSA-based phylogenetic analysis divided the strains in 4 groups, all of which encompassed different levels of genetic diversity. The first clade encompassed all sampled *A. caldus* strains available to us, which happened to pertain exclusively to 16S rRNA tree subclade 1A. Homogeneity of this group of strains from a genomic point of view, was demonstrated previously using species-specific MLSA markers (Nuñez et al., 2014). Notably, none of the strains in our collection was affiliated with the 1B, 1C, or 1D subclades, which are mostly Asiatic or American in origin, and seem to be considerably less common than subclade 1A strains. Strains from the rare subclades that can be traced to natural environments seem to be associated to sulfidic caves (Jones et al., 2016) or geothermal sites (Urbieta et al., 2015). Strains from these subclades are as much as 3.7–5.2% divergent with respect to the type strain of *A. caldus* (ATCC 51756) at the level of the 16S rRNA gene, indicating that they comprise distinct species or even distinct genera. Divergence between *A. caldus* and *A. thiooxidans* is also close to the 5% limit value, generally accepted as boundary to discriminate genera.

According to recent studies, only 10% of the current bacterial species with validly published names conform to the established species or even the genus thresholds (Rossi-Tamisier et al., 2015). These cutoffs were originally established under the assumption that the level of inter-species 16S rRNA gene sequence variation was homogeneous among genera. However, in the light of the variations in the speed of evolution of these genes between phyla (e.g., Clarridge, 2004), their adequacy has been challenged. Our MLSA data support the view that the clade that groups the type strain of *A. caldus* and other 16S rRNA gene-defined subclades, is almost as divergent with respect to the other *Acidithiobacillus* species as it is to *T. tepidarius*. While *T. tepidarius* is 26.4% divergent from *A. caldus*, 27.6% divergent (on average) from the iron-oxidizing acidithiobacilli and 30.3% divergent (on average) from the *A. thiooxidans* clade members, *A. caldus* diverges from all other *Acidithiobacillu*s species by an average of 24%. These results strongly suggest that this whole lineage should be reassigned to a new genus. However, divergence at the 16S rRNA gene level is above the 94% divergence limits identity cutoff generally used to distinguish genera (Yarza et al., 2008). Clearly, additional criteria will need to be considered to further clarify this situation.

While MLSA supported the distinction of clade II and III lineages (represented respectively by the type/reference strains of *A. ferrooxidans* and *A. ferridurans*, and *A. ferriphilus* and *A. ferrivorans*)**, as distinct species, *A. thiooxidans* and *A. albertensis*, with a global divergence lower than 3%, generally considered as threshold value for species distinction, appeared almost indistinguishable. *A. ferridurans* seems to have become distinct from *A. ferrooxidans* quite recently (3.4% divergence at the level of the 8-gene concatenate) and likewise *A. ferrivorans* from *A. ferriphilus* (3.9% divergent), implying some degree of niche-driven specialization or biogeographical isolation. Metadata and targeted metagenomic data suggest that this is indeed the case for the psychrotolerant species, *A. ferrivorans*, which seems to be mostly restricted to environments experiencing extensive periods of cold temperature, for example at high latitudes or altitudes. Further evidence needs to be generated to explain factors driving *A. ferridurans* differentiation from *A. ferrooxidans*. For the moment, metadata are too poor to derive relevant conclusions in this respect, although the greater tolerance of some *A. ferridurans* strains to extreme acidity, and a greater propensity of growth on hydrogen as sole electron donor with respect to *A. ferrooxidans* have been proposed as diagnostic characteristics of the species (Hedrich and Johnson, 2013).

In turn, differentiation of *A. albertensis* from *A. thiooxidans* seems to be ongoing, as suggested by the nucleotide substitution ratios in the protein-coding loci analyzed. At the level of the single gene trees, the dN/dS ratios for the *A. thiooxidans-A. albertensis* clade had values > 1, indicating that positive selection is ongoing in this branch. This statement is also supported by clearly distinguishable oligotypes between the two lineages at the level of the 16S rRNA gene, implying probable ecotype level differentiation (e.g., Sintes et al., 2016). Some obvious phenotypic features between the two lineages support this view. In particular, *A. albertensis* has a bundle of polar flagella that are unique in the genus, while other flagellated bacteria, like *A. thiooxidans*, only have a single polar flagellum (unpublished). Different types of flagellation have been shown to provide diverse advantages under different environmental conditions (Kearns, 2010). A flagellar bundle in *A. albertensis* may add propulsion forces for displacement in viscous environments or enable more efficient spreading along surfaces, as shown in other bacteria (Bubendorfer et al., 2014), which may in turn, convey greater fitness to *A. albertensis* strains under specific mineral leaching conditions. Further efforts to evaluate niche partitions and relative fitness of these two types of mesophilic sulfur-oxidizers need to be carried out to test this hypothesis.

Even if not all new clades represented in the 16S rRNA gene tree were represented in our culture collection, or available to us from our colleagues, a number of them could be tested at higher molecular resolution. MLSA evidence further supported divergence of several of the emergent lineages and currently recognized species, uncovered using the combined 16S rRNA gene and oligotyping strategies. Using the 3% divergence cutoff, lineages represented by *A. thiooxidans*-like strains ATCC 19703 and GG1-14, and the *A. ferrooxidans*-like strain DSM 1927, emerge as new candidate species. Interestingly, strain ATCC 19703 was described as a sulfuric acid-forming bacterium isolated from moist corroded concrete exposed to atmospheres containing H_2_S (Parker, 1945a). Based on morphological, cultural and biochemical properties this strain was considered to be a different species from *A. thiooxidans*, and was designated as *Thiobacillus concretivorus*. This new candidate species was described as one of the relevant members in a microbial succession driving the corrosion of concrete sewers (Parker, 1945b, 1947), and strain ATCC 19703 was accepted as the type strain of the species (Sneath and Skerman, 1966). Common features between “*T. concretivorus”* and *A. thiooxidans* included their capacity to oxidize thiosulfate, elementary sulfur and hydrogen sulfide (Parker and Prisk, 1953). Distinguishing features included the ability of “*T. concretivorus*” to utilize nitrate, in addition to ammonium, as a nitrogen source for growth, to occasionally precipitate sulfur from thiosulfate instead of directly forming sulfuric acid as *A. thiooxidans* (Parker, 1945a) and its higher tolerance to high concentrations of thiosulfate (Parker and Prisk, 1953). In 1957, Vishniac and colleagues questioned the pertinence of these discriminating criteria (Vishniac and Santer, 1957) and after evaluation of the 16S rRNA gene sequence Kelly reassigned all “*T. concretivorus”* strains to *A. thiooxidans* (Kelly and Wood, 2000). According to our molecular analyses, strain ATCC 19703 cannot be distinguished from *A. thiooxidans* on the basis of 16S rRNA gene analysis nor oligotyping. However, MLSA data uncovered a significant divergence (4.8%) between strain ATCC 19703 and the *A. thiooxidans* subclade that exceeds the currently accepted threshold for species differentiation and points to a genetic distinction between the two groups. Further physiological, chemotaxonomic and genomic analysis should be performed on this strain and its close relatives to resolve this issue.

A much clearer distinction was found at the MLSA concatenate level for strain GG1-14, which is as much as 13.3% divergent with respect to *A. thiooxidans*, a level of divergence that is comparable to that existing between *A. ferrooxidans* and *A. ferrivorans*. This strain was isolated from an acidic (pH 1.9; 25°C) pool on the island of Montserrat (W.I.), but no further physiological or cultural data have been obtained so far that hints on its diagnostic characteristics (Atkinson et al., 2000).

A new lineage, possibly representing a new species, was also found among the iron-oxidizing strains. This lineage, called 2B on the basis of the 16S rRNA gene phylogeny and which groups strains DSM 1927 (strain F221) and CF3 among 39 other isolates and sequence clones, was clearly distinguishable from the *A. ferrooxidans* subclade 2A and the *A. ferridurans* subclade 3B, both according to 16S rRNA gene oligotyping and MLSA sequence typing. In agreement with our findings, strain CF3 has been shown to group as a sister branch with respect to the type strain of *A. ferroxidans* in neighbor-joining phylogenetic trees derived from the same molecular marker (Hedrich and Johnson, 2013). However, according to the same study strain DSM 1927 clusters together with the type strain of *A. ferridurans*. Branching order of these strains had earlier been shown to be unstable (Lane et al., 1992). Previous studies have also shown divergence between strain CF3 and *A. ferrooxidans* using rep-PCR (Paulino et al., 2001). At the level of the MLSA concatenate, strains DSM 1927 and CF3 were below the species divergence threshold cutoff with respect to *A. ferrooxidans* and just above the cutoff with respect to *A. ferridurans*, suggesting they comprise an intermediate group to both in a presently unclear state of differentiation from both these species. Further, phylogenomic studies should cast light on this matter and better resolve if this is a case of ongoing or achieved differentiation. There are a number of physiological characteristics support differentiation of strain DSM 1927, which was originally isolated from a uranium mine drainage in Austria, from both *A. ferrooxidans* and *A. ferridurans*. It is tolerant to uranium (up to 2400 ppm; ~10 mM) and, while it shares 85% DNA-DNA hybridization homology to *A. ferrooxidans* strain ATCC 19859 (subclade 2A) and has numerous features in common with this strain, strain DSM 1927 was able to tolerate 65°C for 5 min without losing viability in contrast with strain ATCC 19859 which perished in the process (Harrison, 1982). Other studies have shown that colonies of strain DSM 1927 are differently pigmented (gray colored) during aerobic growth on hydrogen, in contrast with those of *A. ferridurans* ATCC 33020^T^ which were dark brown and *A. ferrooxidans* ATCC 23270^T^ which remained unpigmented (Hedrich and Johnson, 2013).

Other lineages uncovered by the 16S rRNA gene phylogeny and further supported by the oligotyping data, that could not be evaluated by MLSA in this study and that deserve further attention are subclades 1B, 1C, and 1D, branching close to the *A. caldus* type strain, subclade 4B branching as a sister clade of *A. ferrivorans* and subclade 3A branching next to *A. ferridurans*. Although presently mostly occupied by uncultured clones, all these clades have at least one cultured representative and could be targeted for deeper experimental characterization in order to better span the genetic diversity of the *Acidithiobacillus* species complex.

## Conclusions

The hierarchical relationships among members of the genus *Acidithiobacillus*, all of which are part of a single order and a single family, have remained poorly understood in the past. Using molecular systematics approaches and an extensive set of strains and sequence clones from diverse global locations, we have revised the inherent diversity of the acidithiobacilli and reconstructed a robust genus-level phylogeny. Results obtained in this study confirm, at a much wider scale, the inherent diversity of this taxon and support the recognition of the acidithiobacilli as a species complex. These phylogenetic analyses, utilizing different molecular markers and typing approaches, suggest that this species complex includes hitherto unrecognized genera and species, and also ecotypes in the process of differentiation. The availability of genome sequences from a larger number of strains spanning the complex should enable future detailed phylogenomic studies to resolve the evolutionary relationships with a greater degree of precision and gain insight into the factors driving population differentiation in extremely acidic environments.

## Author contributions

RQ and DBJ conceived and supervised the study. HN and AM designed and carried out the bioinformatic analyses. PC, JA, and LA performed the molecular biology experiments. MG prepared and maintained the strains. FI and JC supported the sequence and statistical analyses. All authors analyzed the data. HN, AM, and RQ analyzed and interpreted the data and wrote the paper. All authors read and approved the final manuscript.

## Funding

FONDECYT 1140048 and 3130376. CONICYT Basal CCTE PFB16 and CONICYT and UNAB graduate study fellowships.

### Conflict of interest statement

The authors declare that the research was conducted in the absence of any commercial or financial relationships that could be construed as a potential conflict of interest.
