# Supplementary material for: Molecular Systematics of the Genus Acidithiobacillus: Insights into the Phylogenetic Structure and Diversification of the Taxon
Source: Front Microbiol. 2017 Jan 19;8:30. doi: 10.3389/fmicb.2017.00030 (PMC5243848; doi:10.3389/fmicb.2017.00030)
Supplement: Supplementary file 7 [file Image1.pdf]

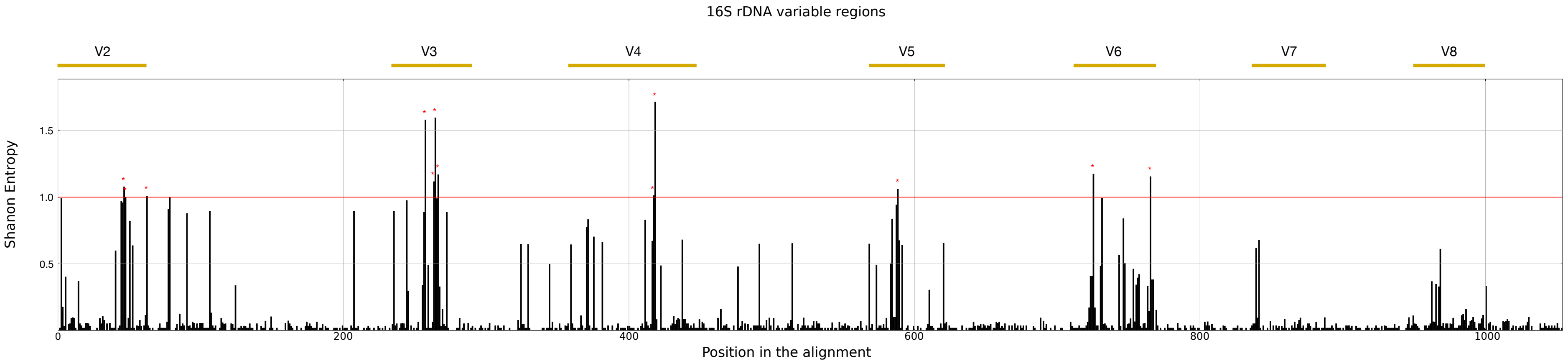

16S rDNA variable regions
V2
V3
V4
V5
V6
V7
V8
Shanon Entropy
1.5
1.0
0.5
0
200
400
600
800
1000
Position in the alignment

Supplementary Figure 1. Shannon entropy analysis of the alignment of the 16S rDNA sequences from 580 *Acidithiobacillus* strains and sequence clones, showing the positions of highest entropy. Variable regions of the *E. coli* 16S rRNA gene are mapped along the sequence as reference.
