# Supplementary material for: Molecular Systematics of the Genus Acidithiobacillus: Insights into the Phylogenetic Structure and Diversification of the Taxon
Source: Front Microbiol. 2017 Jan 19;8:30. doi: 10.3389/fmicb.2017.00030 (PMC5243848; doi:10.3389/fmicb.2017.00030)
Supplement: Supplementary file 8 [file Image2.pdf]

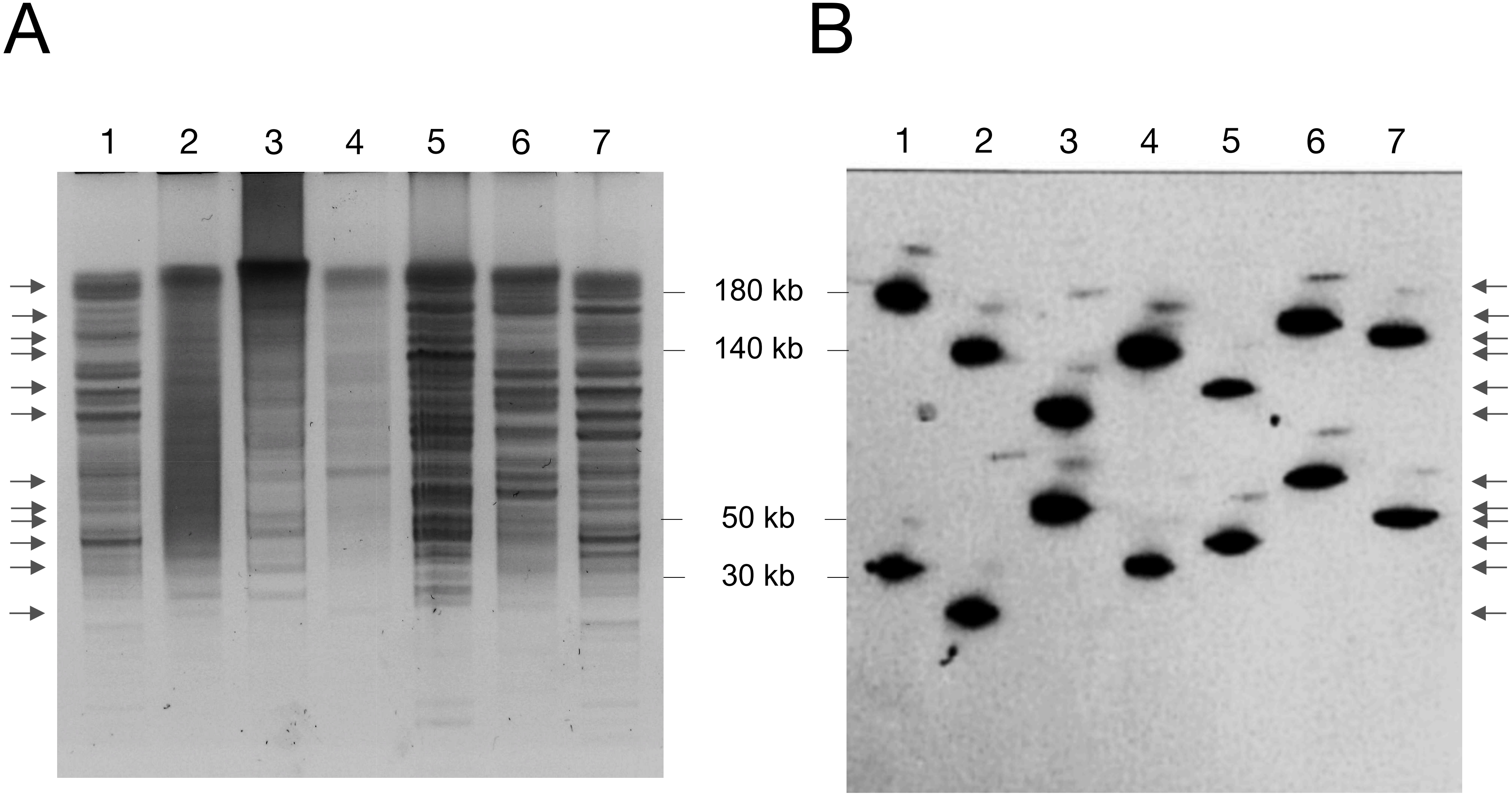
A
B
1 2 3 4 5 6 7
1 2 3 4 5 6 7
180 kb
140 kb
50 kb
30 kb

Supplementary Figure 2. Ribosomal RNA operon copy number in *Acidithiobacillus* spp. (**A**) PFGE of genomic DNA digested with HpaI for reference strains. (**B**) Southern blot with a probe targeting an internal fragment of the 16S rRNA gene. DNA marker size ranges are indicated between panels.

Strains are displayed in the following order: (1) *A. ferrooxidans*^{T} ATCC 23270, (2) *A. ferridurans*^{T} ATCC 33020, (3) *A. ferrivorans* PQ510, (4) *A. thiooxidans*^{T} ATCC 19377, (5) *A. thiooxidans* GG1-14, (6) *A. albertensis*^{T} DSM 14366, (7) *A. caldus*^{T} ATCC 51756.
