# Supplementary material for: Molecular Systematics of the Genus Acidithiobacillus: Insights into the Phylogenetic Structure and Diversification of the Taxon
Source: Front Microbiol. 2017 Jan 19;8:30. doi: 10.3389/fmicb.2017.00030 (PMC5243848; doi:10.3389/fmicb.2017.00030)
Supplement: Supplementary file 9 [file Image3.PDF]

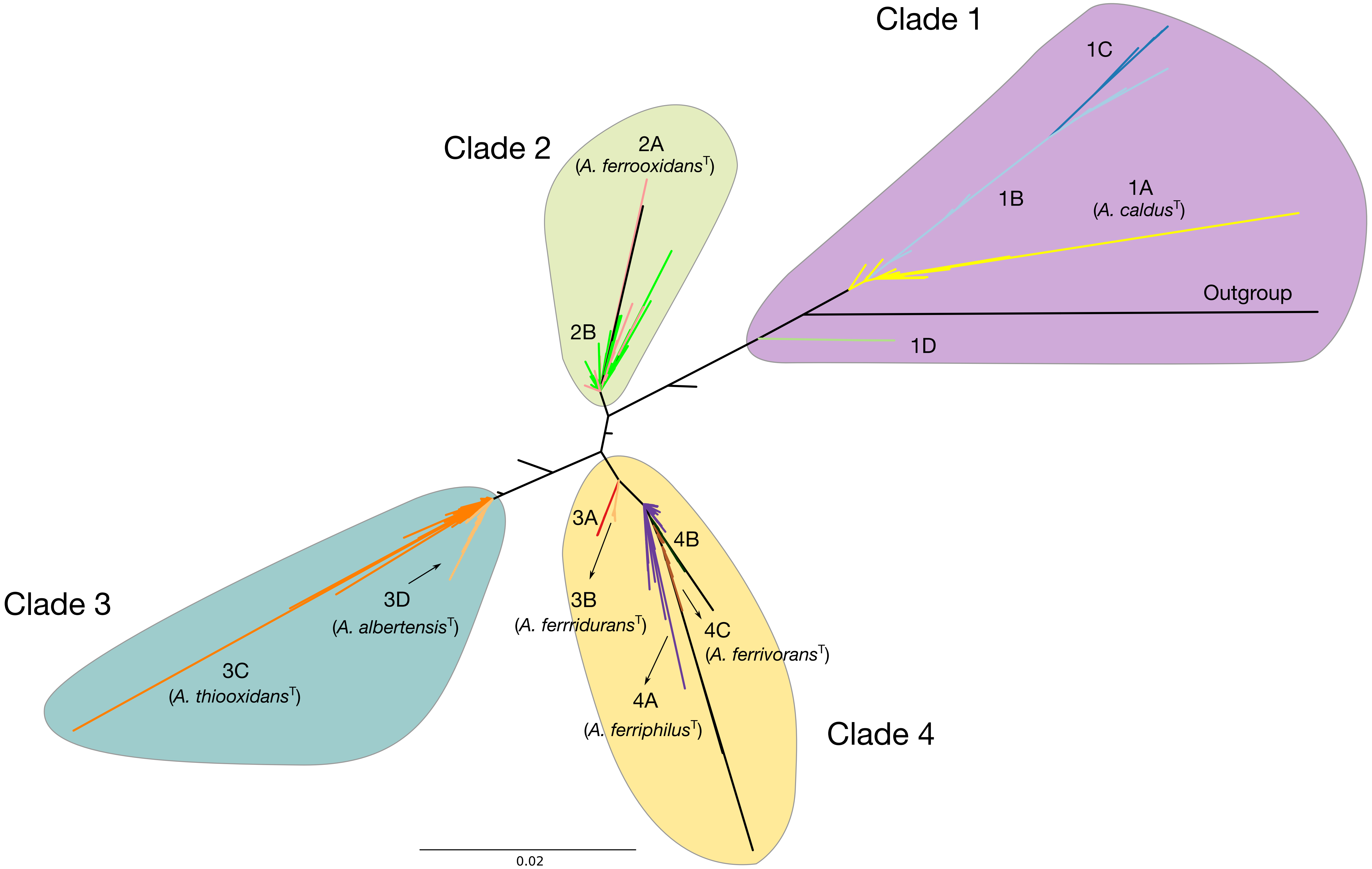

Clade 1
1C
1B
1A
(A. caldusT)
Outgroup
1D
Clade 2
2A
(A. ferrooxidansT)
2B
Clade 3
3D
(A. albertensisT)
3C
(A. thiooxidansT)
3A
3B
(A. ferriduransT)
4B
4C
(A. ferrivoransT)
4A
(A. ferriphilusT)
Clade 4
0.02

Supplementary Figure 3. *Acidithiobacillus* species complex consensus phylogenetic tree built using Bayesian inference and 16S rRNA gene sequences of 580 strains and/or sequence clones. Clades affiliations are as follows: clade 1 (purple, *A. caldus*^{T}), clade 2 (green, *A. ferrooxidans*^{T}), clade 3 (turquoise, *A. thiooxidans*^{T}, *A. albertensis*^{T}), clade 4 (yellow, *A. ferriphilus*^{T}, *A. ferrivorans*^{T}, *A. ferridurans*^{T}). Subclades 1A through 4C are color coded according to the oligotype (OT) assigned to the strains and sequence clones that conform each cluster.
