# Supplementary material for: Molecular Systematics of the Genus Acidithiobacillus: Insights into the Phylogenetic Structure and Diversification of the Taxon
Source: Front Microbiol. 2017 Jan 19;8:30. doi: 10.3389/fmicb.2017.00030 (PMC5243848; doi:10.3389/fmicb.2017.00030)
Supplement: Supplementary file 10 [file Image4.PDF]

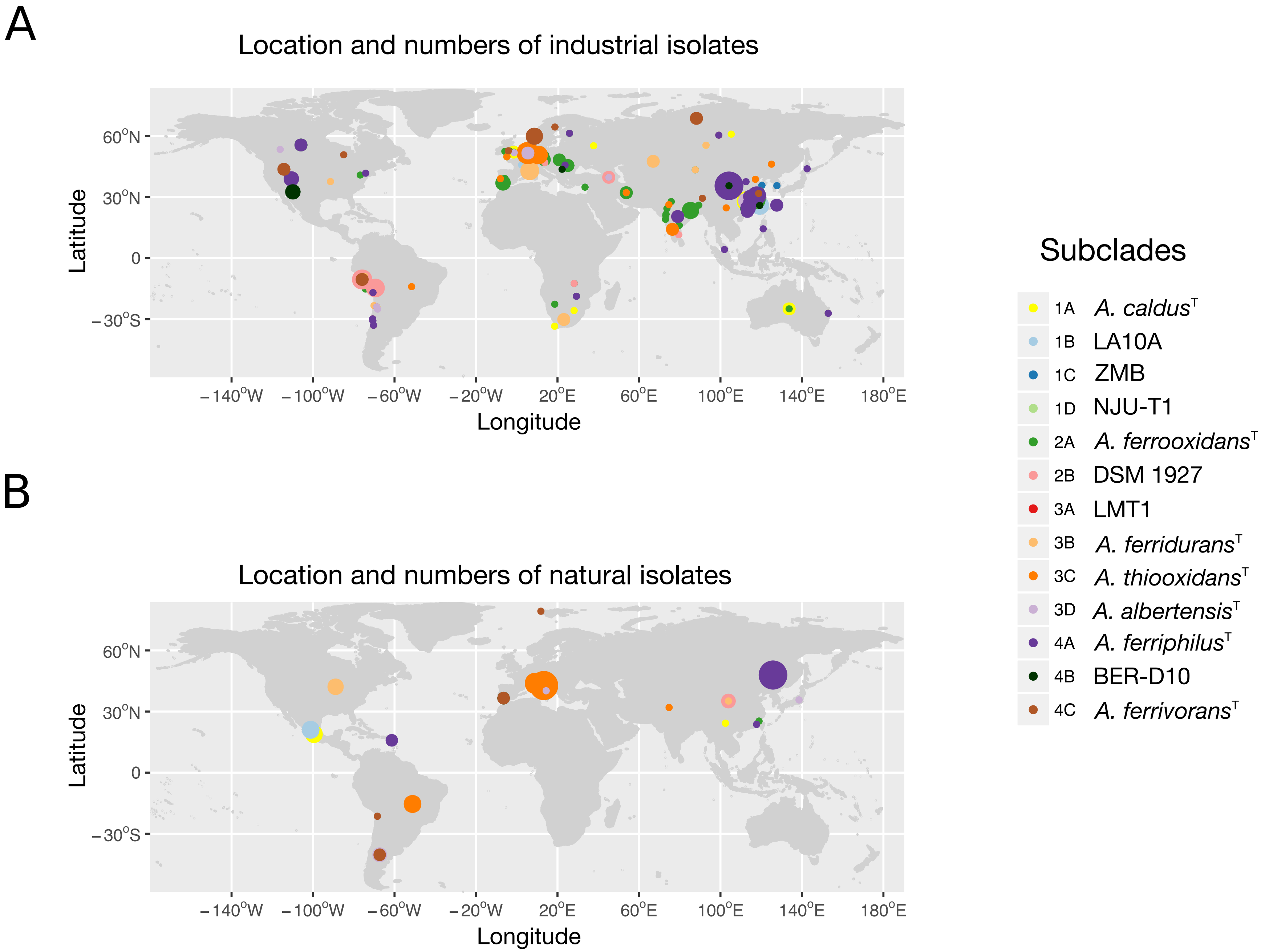
A
Location and numbers of industrial isolates
60°N
30°N
0
−30°S
Latitude
−140°W
−100°W
−60°W
−20°W
20°E
60°E
100°E
140°E
180°E
Longitude
B
Location and numbers of natural isolates
60°N
30°N
0
−30°S
Latitude
−140°W
−100°W
−60°W
−20°W
20°E
60°E
100°E
140°E
180°E
Longitude
Subclades
1A A. caldusT
1B LA10A
1C ZMB
1D NJU-T1
2A A. ferrooxidansT
2B DSM 1927
3A LMT1
3B A. ferriduransT
3C A. thiooxidansT
3D A. albertensisT
4A A. ferriphilusT
4B BER-D10
4C A. ferrivoransT

Supplementary Figure 4. Occurrence and prevalence of the *Acidithiobacillus* species complex subclades in acidic econiches around the globe in (**A**) industrial and (**B**) natural settings. Detailed information of the location and type of setting for each of the strains mapped can be found in Supplementary Table 3.
