# Supplementary material for: Molecular Systematics of the Genus Acidithiobacillus: Insights into the Phylogenetic Structure and Diversification of the Taxon
Source: Front Microbiol. 2017 Jan 19;8:30. doi: 10.3389/fmicb.2017.00030 (PMC5243848; doi:10.3389/fmicb.2017.00030)
Supplement: Supplementary file 11 [file Image5.PDF]

A)

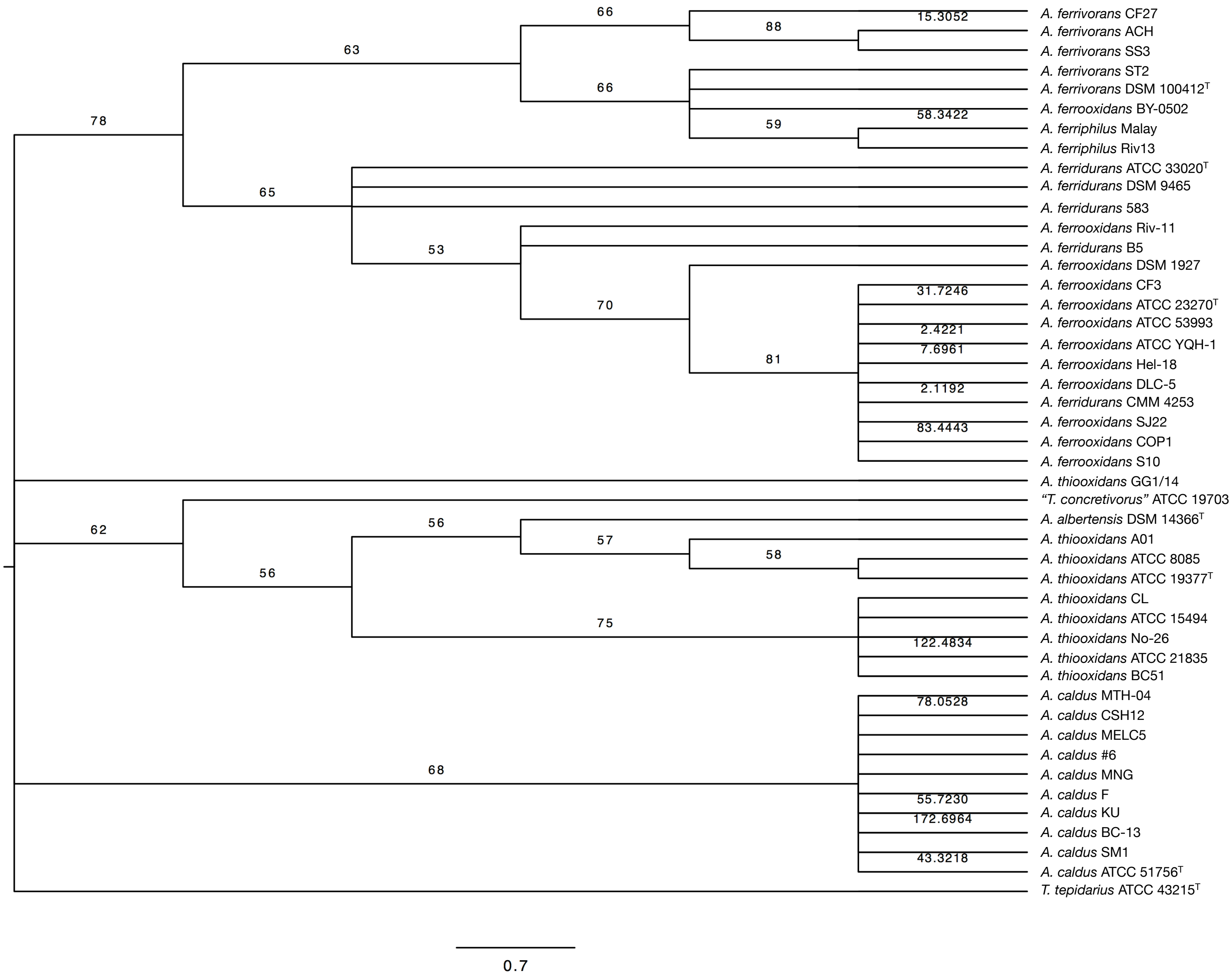

A. ferrivorans CF27
A. ferrivorans ACH
A. ferrivorans SS3
A. ferrivorans ST2
A. ferrivorans DSM 100412T
A. ferrooxidans BY-0502
A. ferriphilus Malay
A. ferriphilus Riv13
A. ferridurans ATCC 33020T
A. ferridurans DSM 9465
A. ferridurans 583
A. ferrooxidans Riv-11
A. ferridurans B5
A. ferrooxidans DSM 1927
A. ferrooxidans CF3
A. ferrooxidans ATCC 23270T
A. ferrooxidans ATCC 53993
A. ferrooxidans ATCC YQH-1
A. ferrooxidans Hel-18
A. ferrooxidans DLC-5
A. ferridurans CMM 4253
A. ferrooxidans SJ22
A. ferrooxidans COP1
A. ferrooxidans S10
A. thiooxidans GG1/14
"T. concretivorus" ATCC 19703
A. albertensis DSM 14366T
A. thiooxidans A01
A. thiooxidans ATCC 8085
A. thiooxidans ATCC 19377T
A. thiooxidans CL
A. thiooxidans ATCC 15494
A. thiooxidans No-26
A. thiooxidans ATCC 21835
A. thiooxidans BC51
A. caldus MTH-04
A. caldus CSH12
A. caldus MELC5
A. caldus #6
A. caldus MNG
A. caldus F
A. caldus KU
A. caldus BC-13
A. caldus SM1
A. caldus ATCC 51756T
T. tepidarius ATCC 43215T
66
88
15.3052
63
66
59
58.3422
78
65
53
70
81
31.7246
2.4221
7.6961
2.1192
83.4443
62
56
57
58
56
75
122.4834
68
78.0528
55.7230
172.6964
43.3218
0.7

B)

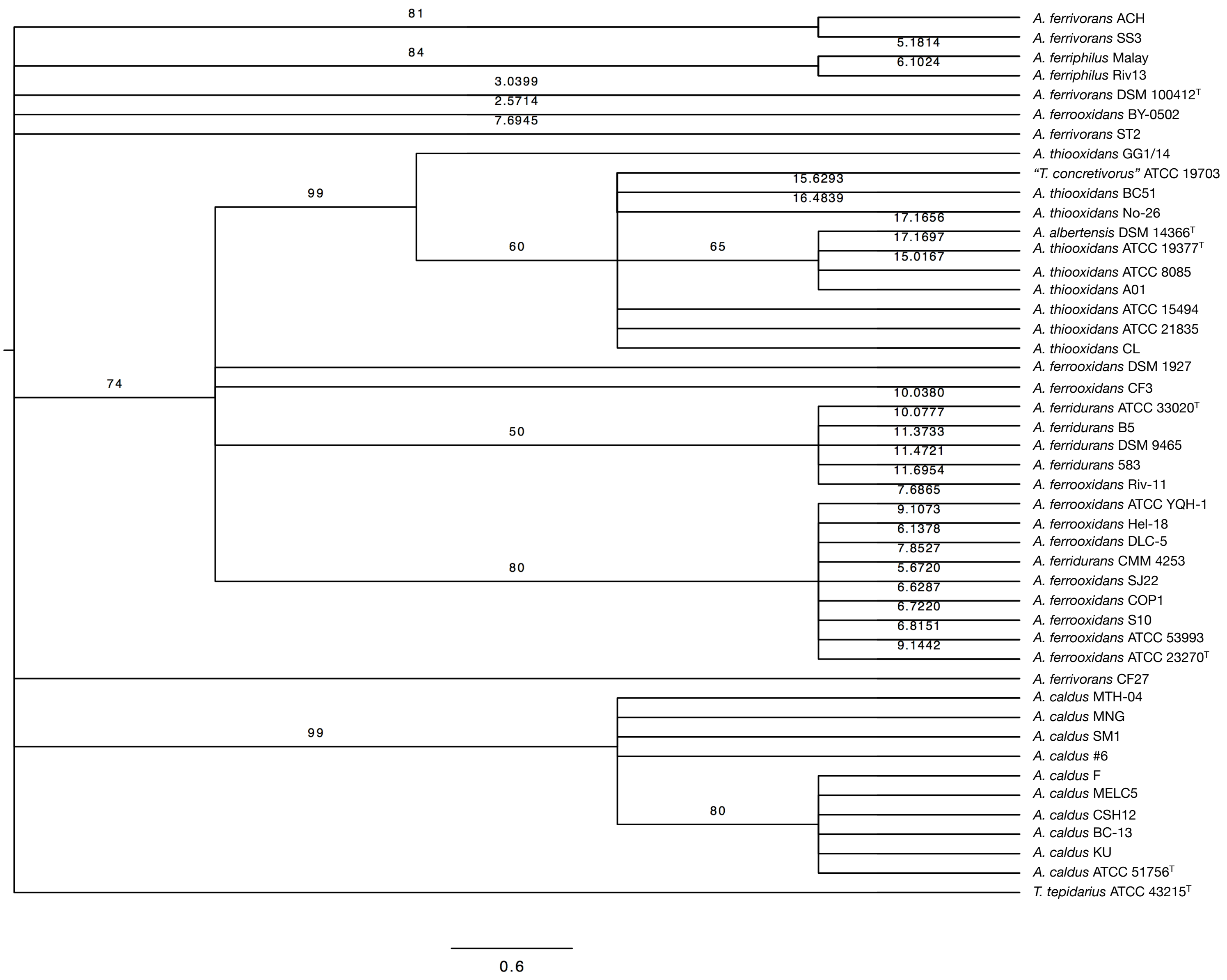

81
84
5.1814
6.1024
3.0399
2.5714
7.6945
99
15.6293
16.4839
17.1656
17.1697
15.0167
60
65
74
10.0380
10.0777
11.3733
11.4721
11.6954
7.6865
9.1073
6.1378
7.8527
5.6720
6.6287
6.7220
6.8151
9.1442
50
80
99
80
A. ferrivorans ACH
A. ferrivorans SS3
A. ferriphilus Malay
A. ferriphilus Riv13
A. ferrivorans DSM 100412T
A. ferrooxidans BY-0502
A. ferrivorans ST2
A. thiooxidans GG1/14
“T. concretivorus” ATCC 19703
A. thiooxidans BC51
A. thiooxidans No-26
A. albertensis DSM 14366T
A. thiooxidans ATCC 19377T
A. thiooxidans ATCC 8085
A. thiooxidans A01
A. thiooxidans ATCC 15494
A. thiooxidans ATCC 21835
A. thiooxidans CL
A. ferrooxidans DSM 1927
A. ferrooxidans CF3
A. ferridurans ATCC 33020T
A. ferridurans B5
A. ferridurans DSM 9465
A. ferridurans 583
A. ferrooxidans Riv-11
A. ferrooxidans ATCC YQH-1
A. ferrooxidans Hel-18
A. ferrooxidans DLC-5
A. ferridurans CMM 4253
A. ferrooxidans SJ22
A. ferrooxidans COP1
A. ferrooxidans S10
A. ferrooxidans ATCC 53993
A. ferrooxidans ATCC 23270T
A. ferrivorans CF27
A. caldus MTH-04
A. caldus MNG
A. caldus SM1
A. caldus #6
A. caldus F
A. caldus MELC5
A. caldus CSH12
A. caldus BC-13
A. caldus KU
A. caldus ATCC 51756T
T. tepidarius ATCC 43215T
0.6

C)

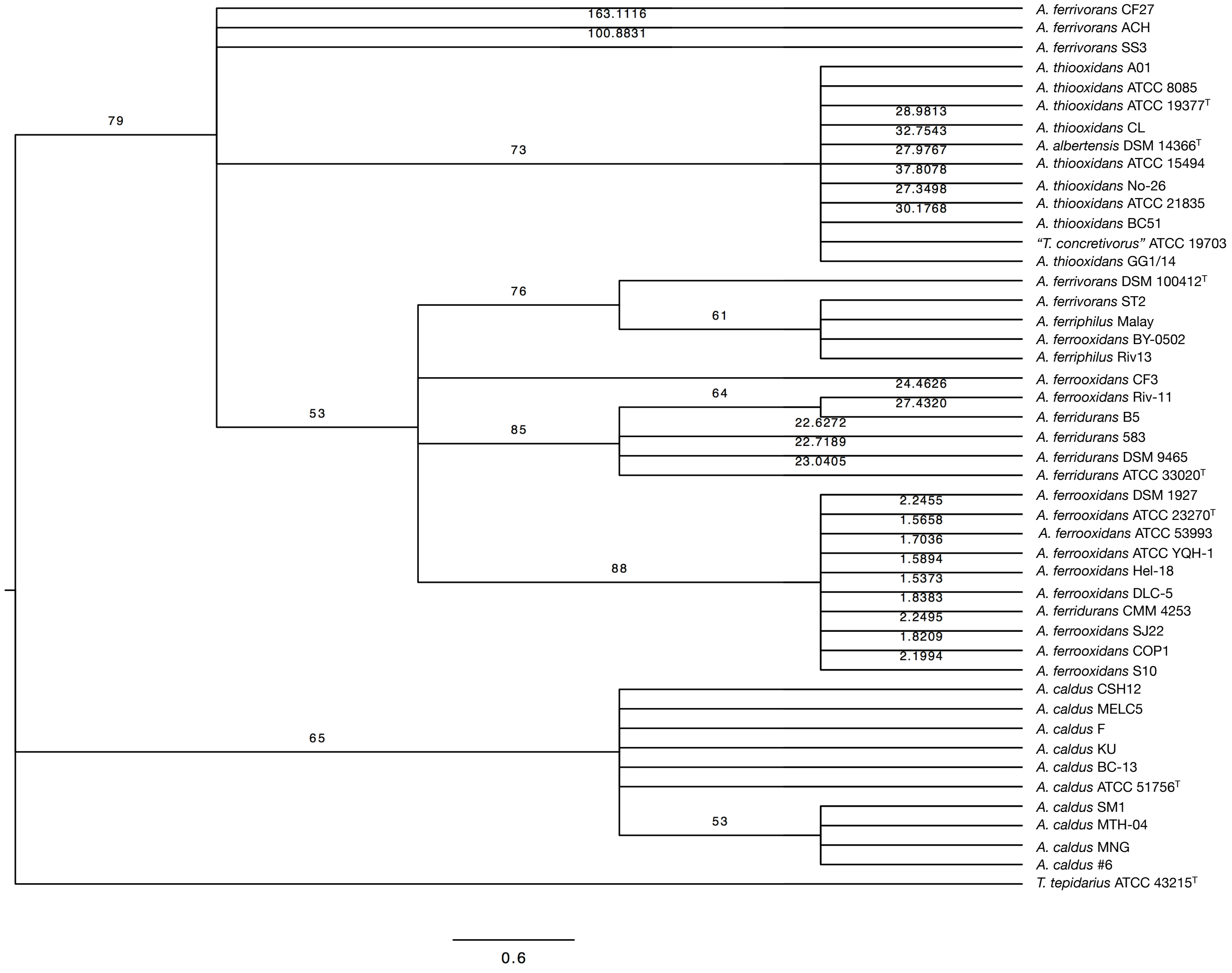
A. ferrivorans CF27
A. ferrivorans ACH
A. ferrivorans SS3
A. thiooxidans A01
A. thiooxidans ATCC 8085
A. thiooxidans ATCC 19377T
A. thiooxidans CL
A. albertensis DSM 14366T
A. thiooxidans ATCC 15494
A. thiooxidans No-26
A. thiooxidans ATCC 21835
A. thiooxidans BC51
"T. concretivorus" ATCC 19703
A. thiooxidans GG1/14
A. ferrivorans DSM 100412T
A. ferrivorans ST2
A. ferriphilus Malay
A. ferrooxidans BY-0502
A. ferriphilus Riv13
A. ferrooxidans CF3
A. ferrooxidans Riv-11
A. ferridurans B5
A. ferridurans 583
A. ferridurans DSM 9465
A. ferridurans ATCC 33020T
A. ferrooxidans DSM 1927
A. ferrooxidans ATCC 23270T
A. ferrooxidans ATCC 53993
A. ferrooxidans ATCC YQH-1
A. ferrooxidans Hel-18
A. ferrooxidans DLC-5
A. ferridurans CMM 4253
A. ferrooxidans SJ22
A. ferrooxidans COP1
A. ferrooxidans S10
A. caldus CSH12
A. caldus MELC5
A. caldus F
A. caldus KU
A. caldus BC-13
A. caldus ATCC 51756T
A. caldus SM1
A. caldus MTH-04
A. caldus MNG
A. caldus #6
T. tepidarius ATCC 43215T
163.1116
100.8831
79
73
28.9813
32.7543
27.9767
37.8078
27.3498
30.1768
76
61
53
24.4626
27.4320
64
85
22.6272
22.7189
23.0405
88
2.2455
1.5658
1.7036
1.5894
1.5373
1.8383
2.2495
1.8209
2.1994
65
53
0.6

D)

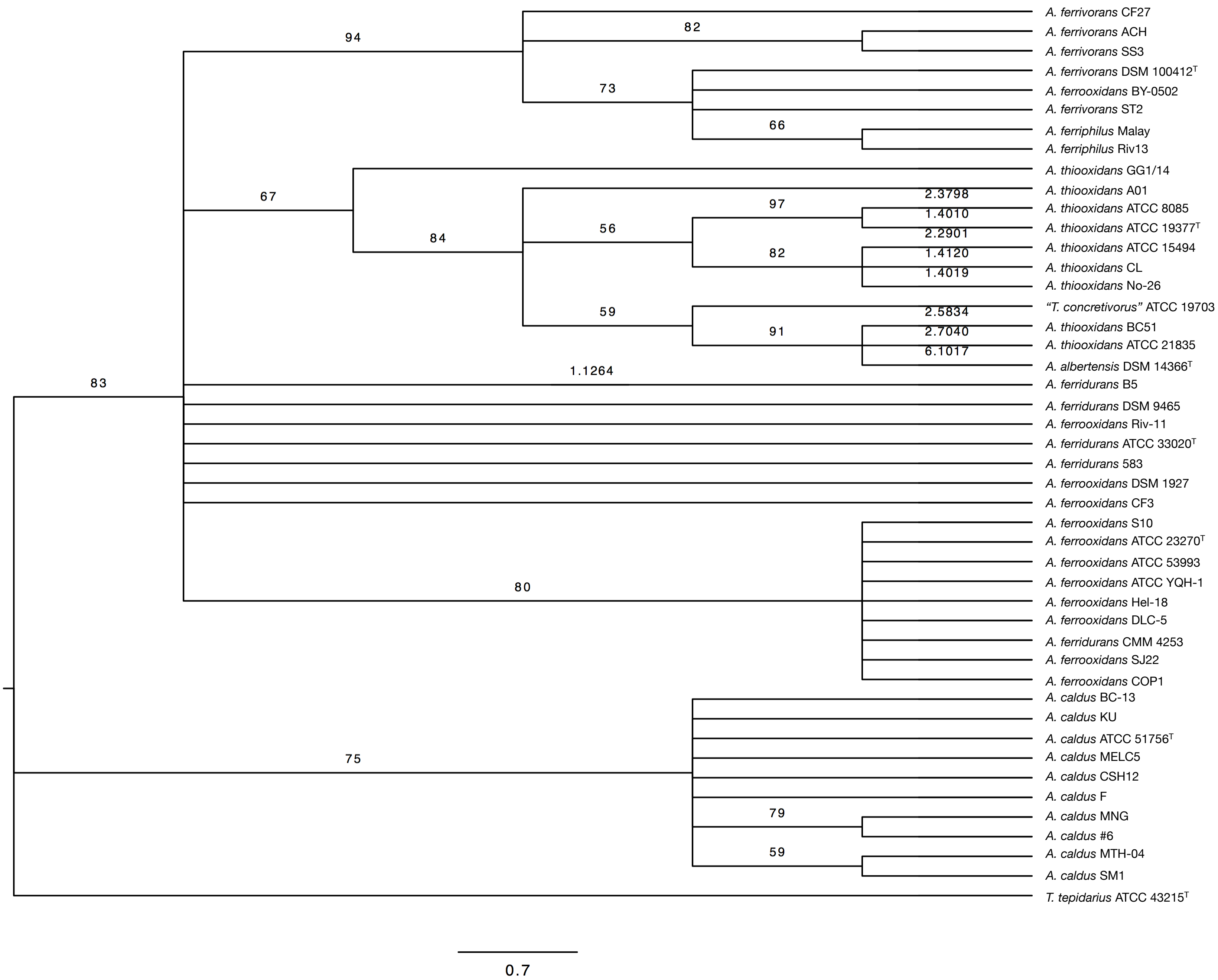
A. ferrivorans CF27
A. ferrivorans ACH
A. ferrivorans SS3
A. ferrivorans DSM 100412T
A. ferrooxidans BY-0502
A. ferrivorans ST2
A. ferriphilus Malay
A. ferriphilus Riv13
A. thiooxidans GG1/14
A. thiooxidans A01
A. thiooxidans ATCC 8085
A. thiooxidans ATCC 19377T
A. thiooxidans ATCC 15494
A. thiooxidans CL
A. thiooxidans No-26
"T. concretivorus" ATCC 19703
A. thiooxidans BC51
A. thiooxidans ATCC 21835
A. albertensis DSM 14366T
A. ferridurans B5
A. ferridurans DSM 9465
A. ferrooxidans Riv-11
A. ferridurans ATCC 33020T
A. ferridurans 583
A. ferrooxidans DSM 1927
A. ferrooxidans CF3
A. ferrooxidans S10
A. ferrooxidans ATCC 23270T
A. ferrooxidans ATCC 53993
A. ferrooxidans ATCC YQH-1
A. ferrooxidans Hel-18
A. ferrooxidans DLC-5
A. ferridurans CMM 4253
A. ferrooxidans SJ22
A. ferrooxidans COP1
A. caldus BC-13
A. caldus KU
A. caldus ATCC 51756T
A. caldus MELC5
A. caldus CSH12
A. caldus F
A. caldus MNG
A. caldus #6
A. caldus MTH-04
A. caldus SM1
T. tepidarius ATCC 43215T
94
82
73
66
67
84
97
56
82
59
91
2.3798
1.4010
2.2901
1.4120
1.4019
2.5834
2.7040
6.1017
1.1264
83
80
75
79
59
0.7

E)

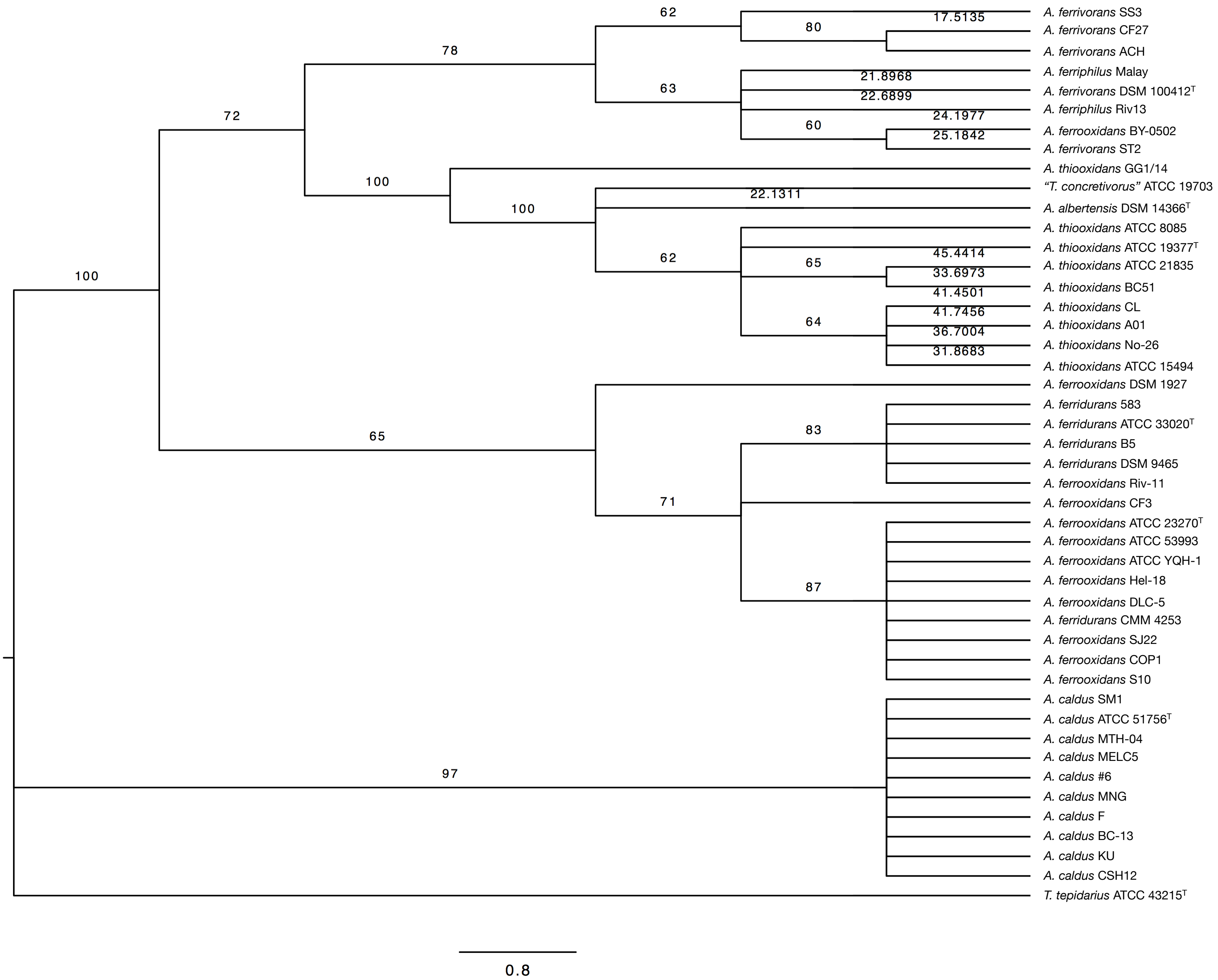

62
80
17.5135
78
72
63
21.8968
22.6899
24.1977
60
25.1842
100
100
22.1311
62
65
45.4414
33.6973
41.4501
64
41.7456
36.7004
31.8683
100
65
83
71
87
97
0.8
A. ferrivorans SS3
A. ferrivorans CF27
A. ferrivorans ACH
A. ferriphilus Malay
A. ferrivorans DSM 100412T
A. ferriphilus Riv13
A. ferrooxidans BY-0502
A. ferrivorans ST2
A. thiooxidans GG1/14
"T. concretivorus" ATCC 19703
A. albertensis DSM 14366T
A. thiooxidans ATCC 8085
A. thiooxidans ATCC 19377T
A. thiooxidans ATCC 21835
A. thiooxidans BC51
A. thiooxidans CL
A. thiooxidans A01
A. thiooxidans No-26
A. thiooxidans ATCC 15494
A. ferrooxidans DSM 1927
A. ferridurans 583
A. ferridurans ATCC 33020T
A. ferridurans B5
A. ferridurans DSM 9465
A. ferrooxidans Riv-11
A. ferrooxidans CF3
A. ferrooxidans ATCC 23270T
A. ferrooxidans ATCC 53993
A. ferrooxidans ATCC YQH-1
A. ferrooxidans Hel-18
A. ferrooxidans DLC-5
A. ferridurans CMM 4253
A. ferrooxidans SJ22
A. ferrooxidans COP1
A. ferrooxidans S10
A. caldus SM1
A. caldus ATCC 51756T
A. caldus MTH-04
A. caldus MELC5
A. caldus #6
A. caldus MNG
A. caldus F
A. caldus BC-13
A. caldus KU
A. caldus CSH12
T. tepidarius ATCC 43215T

F)

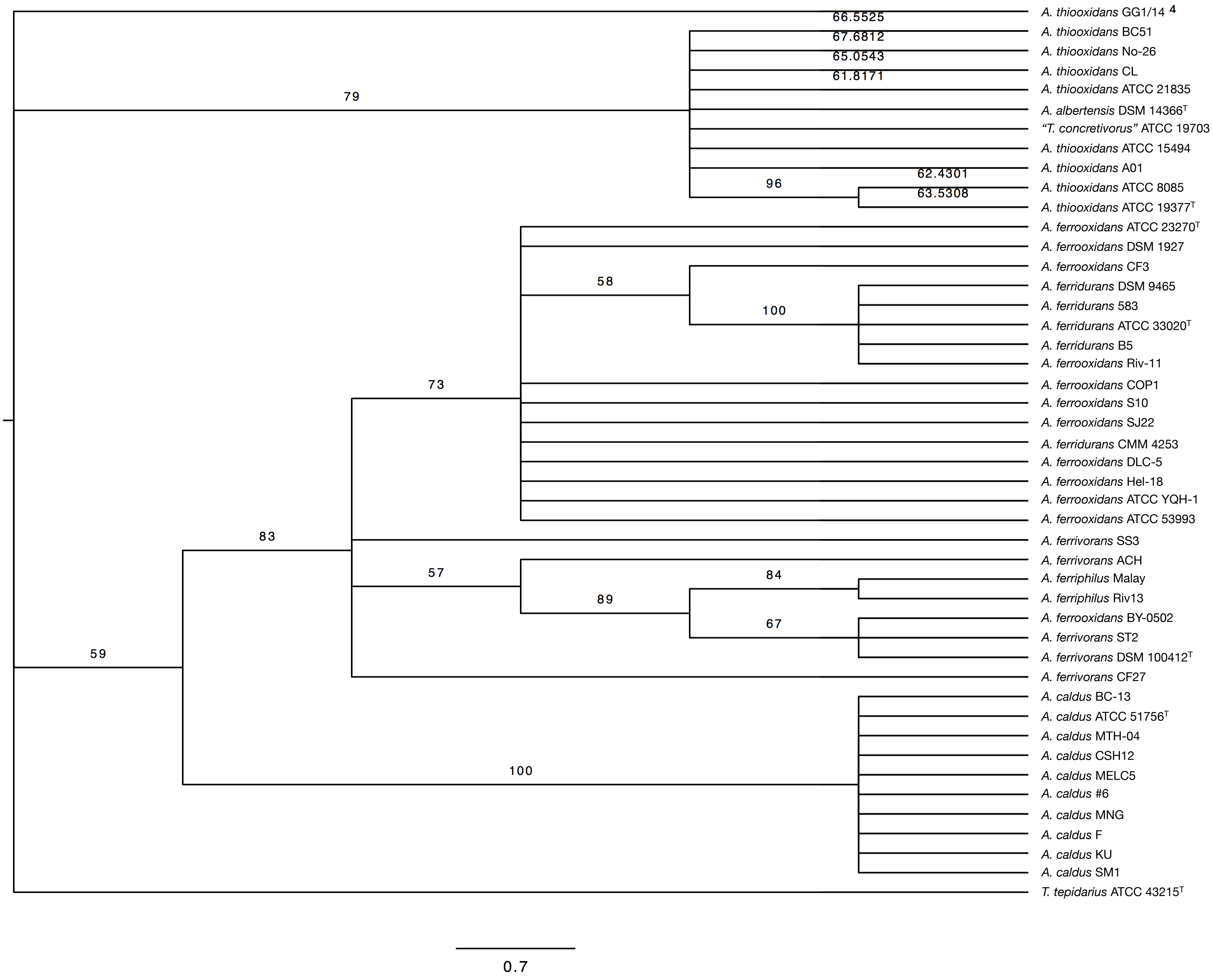

A. thiooxidans GG1/14 4
66.5525
A. thiooxidans BC51
67.6812
A. thiooxidans No-26
65.0543
A. thiooxidans CL
61.8171
A. thiooxidans ATCC 21835
79
A. albertensis DSM 14366T
"T. concretivorus" ATCC 19703
A. thiooxidans ATCC 15494
A. thiooxidans A01
96
62.4301
A. thiooxidans ATCC 8085
63.5308
A. thiooxidans ATCC 19377T
A. ferrooxidans ATCC 23270T
A. ferrooxidans DSM 1927
58
A. ferrooxidans CF3
A. ferridurans DSM 9465
100
A. ferridurans 583
A. ferridurans ATCC 33020T
A. ferridurans B5
A. ferrooxidans Riv-11
73
A. ferrooxidans COP1
A. ferrooxidans S10
A. ferrooxidans SJ22
A. ferridurans CMM 4253
A. ferrooxidans DLC-5
A. ferrooxidans Hel-18
A. ferrooxidans ATCC YQH-1
A. ferrooxidans ATCC 53993
83
A. ferrivorans SS3
57
A. ferrivorans ACH
84
A. ferriphilus Malay
89
A. ferriphilus Riv13
67
A. ferrooxidans BY-0502
A. ferrivorans ST2
59
A. ferrivorans DSM 100412T
A. ferrivorans CF27
A. caldus BC-13
A. caldus ATCC 51756T
A. caldus MTH-04
A. caldus CSH12
100
A. caldus MELC5
A. caldus #6
A. caldus MNG
A. caldus F
A. caldus KU
A. caldus SM1
T. tepidarius ATCC 43215T
0.7

G)

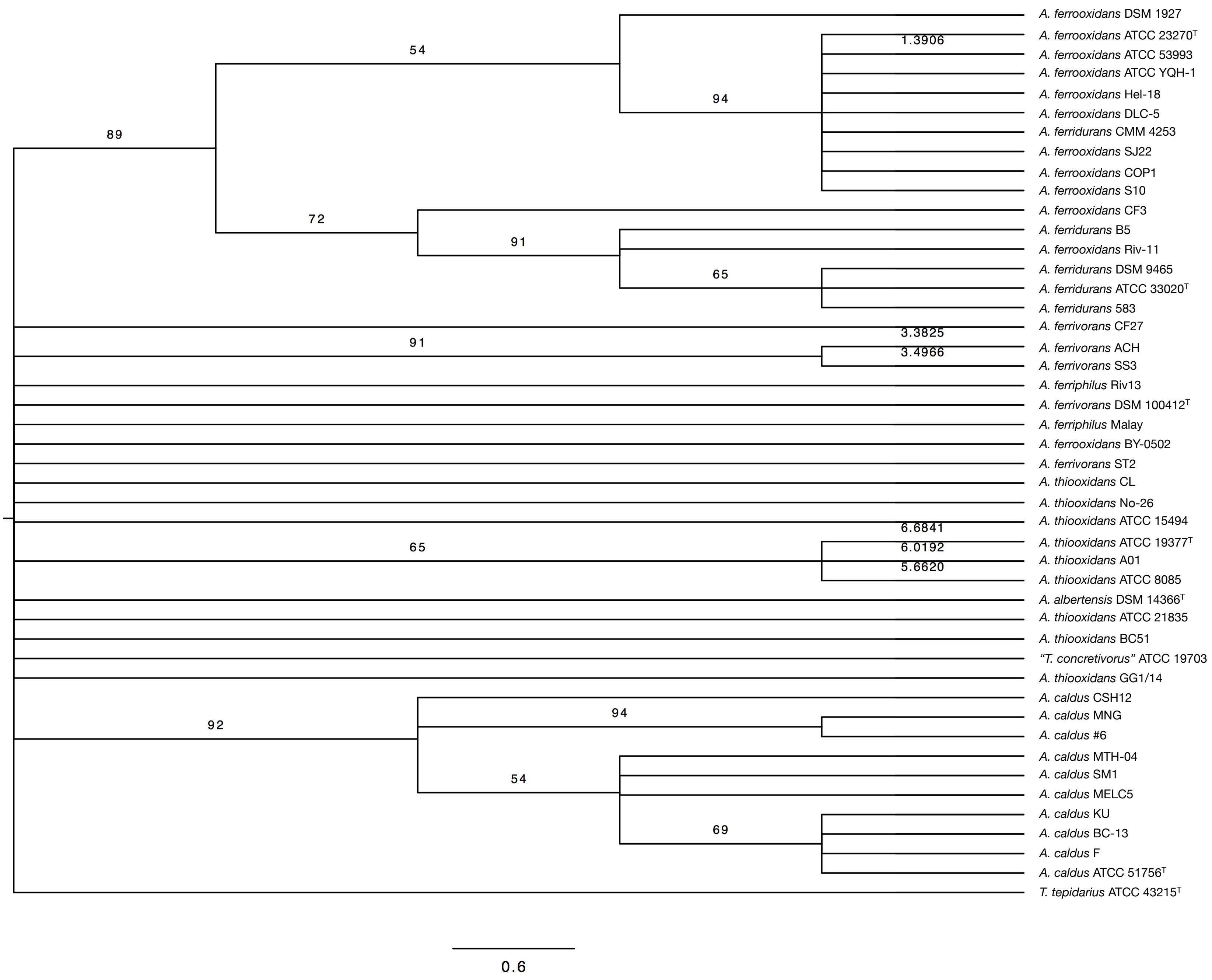
A. ferrooxidans DSM 1927
A. ferrooxidans ATCC 23270T
A. ferrooxidans ATCC 53993
A. ferrooxidans ATCC YQH-1
A. ferrooxidans Hel-18
A. ferrooxidans DLC-5
A. ferridurans CMM 4253
A. ferrooxidans SJ22
A. ferrooxidans COP1
A. ferrooxidans S10
A. ferrooxidans CF3
A. ferridurans B5
A. ferrooxidans Riv-11
A. ferridurans DSM 9465
A. ferridurans ATCC 33020T
A. ferridurans 583
A. ferrivorans CF27
A. ferrivorans ACH
A. ferrivorans SS3
A. ferriphilus Riv13
A. ferrivorans DSM 100412T
A. ferriphilus Malay
A. ferrooxidans BY-0502
A. ferrivorans ST2
A. thiooxidans CL
A. thiooxidans No-26
A. thiooxidans ATCC 15494
A. thiooxidans ATCC 19377T
A. thiooxidans A01
A. thiooxidans ATCC 8085
A. albertensis DSM 14366T
A. thiooxidans ATCC 21835
A. thiooxidans BC51
“T. concretivorus” ATCC 19703
A. thiooxidans GG1/14
A. caldus CSH12
A. caldus MNG
A. caldus #6
A. caldus MTH-04
A. caldus SM1
A. caldus MELC5
A. caldus KU
A. caldus BC-13
A. caldus F
A. caldus ATCC 51756T
T. tepidarius ATCC 43215T
54
89
94
1.3906
72
91
65
91
3.3825
3.4966
65
6.6841
6.0192
5.6620
92
94
54
69
0.6

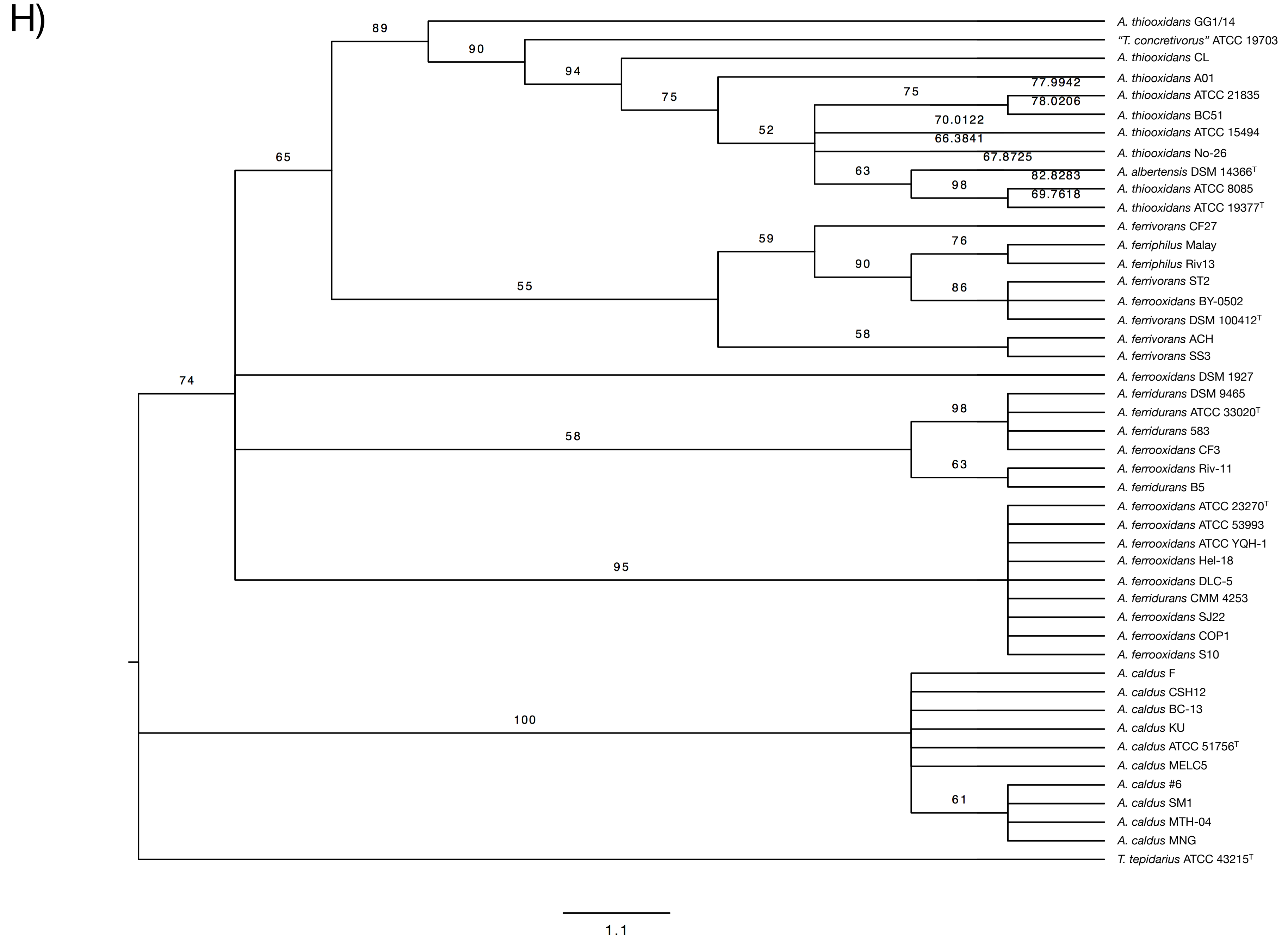
H)
89
90
94
75
52
75
63
98
65
59
76
90
86
55
58
74
58
98
63
95
100
61
77.9942
78.0206
70.0122
66.3841
67.8725
82.8283
69.7618
A. thiooxidans GG1/14
"T. concretivorus" ATCC 19703
A. thiooxidans CL
A. thiooxidans A01
A. thiooxidans ATCC 21835
A. thiooxidans BC51
A. thiooxidans ATCC 15494
A. thiooxidans No-26
A. albertensis DSM 14366T
A. thiooxidans ATCC 8085
A. thiooxidans ATCC 19377T
A. ferrivorans CF27
A. ferriphilus Malay
A. ferriphilus Riv13
A. ferrivorans ST2
A. ferrooxidans BY-0502
A. ferrivorans DSM 100412T
A. ferrivorans ACH
A. ferrivorans SS3
A. ferrooxidans DSM 1927
A. ferridurans DSM 9465
A. ferridurans ATCC 33020T
A. ferridurans 583
A. ferrooxidans CF3
A. ferrooxidans Riv-11
A. ferridurans B5
A. ferrooxidans ATCC 23270T
A. ferrooxidans ATCC 53993
A. ferrooxidans ATCC YQH-1
A. ferrooxidans Hel-18
A. ferrooxidans DLC-5
A. ferridurans CMM 4253
A. ferrooxidans SJ22
A. ferrooxidans COP1
A. ferrooxidans S10
A. caldus F
A. caldus CSH12
A. caldus BC-13
A. caldus KU
A. caldus ATCC 51756T
A. caldus MELC5
A. caldus #6
A. caldus SM1
A. caldus MTH-04
A. caldus MNG
T. tepidarius ATCC 43215T
1.1

I)

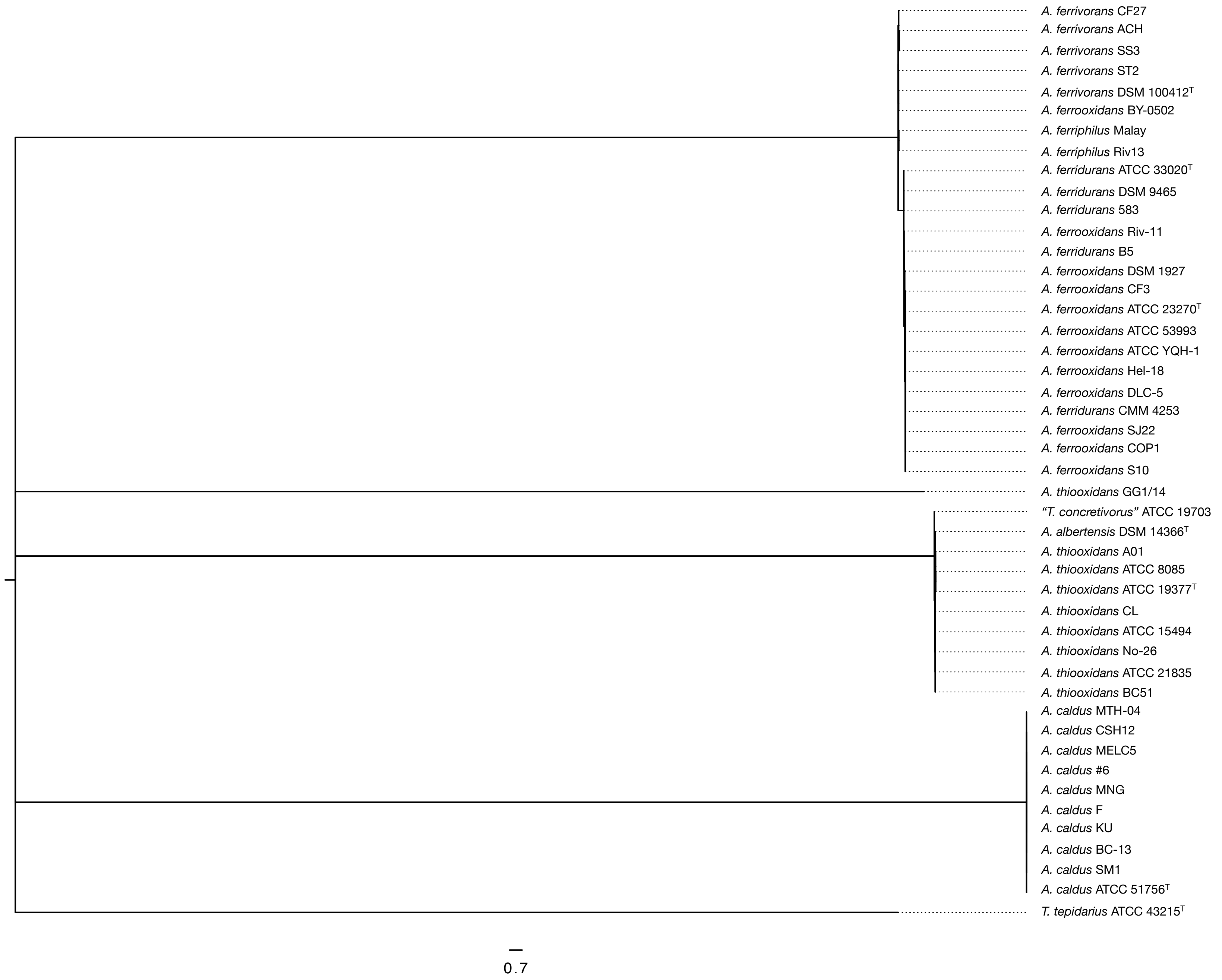
A. ferrivorans CF27
A. ferrivorans ACH
A. ferrivorans SS3
A. ferrivorans ST2
A. ferrivorans DSM 100412T
A. ferrooxidans BY-0502
A. ferriphilus Malay
A. ferriphilus Riv13
A. ferridurans ATCC 33020T
A. ferridurans DSM 9465
A. ferridurans 583
A. ferrooxidans Riv-11
A. ferridurans B5
A. ferrooxidans DSM 1927
A. ferrooxidans CF3
A. ferrooxidans ATCC 23270T
A. ferrooxidans ATCC 53993
A. ferrooxidans ATCC YQH-1
A. ferrooxidans Hel-18
A. ferrooxidans DLC-5
A. ferridurans CMM 4253
A. ferrooxidans SJ22
A. ferrooxidans COP1
A. ferrooxidans S10
A. thiooxidans GG1/14
"T. concretivorus" ATCC 19703
A. albertensis DSM 14366T
A. thiooxidans A01
A. thiooxidans ATCC 8085
A. thiooxidans ATCC 19377T
A. thiooxidans CL
A. thiooxidans ATCC 15494
A. thiooxidans No-26
A. thiooxidans ATCC 21835
A. thiooxidans BC51
A. caldus MTH-04
A. caldus CSH12
A. caldus MELC5
A. caldus #6
A. caldus MNG
A. caldus F
A. caldus KU
A. caldus BC-13
A. caldus SM1
A. caldus ATCC 51756T
T. tepidarius ATCC 43215T
0.7

J)

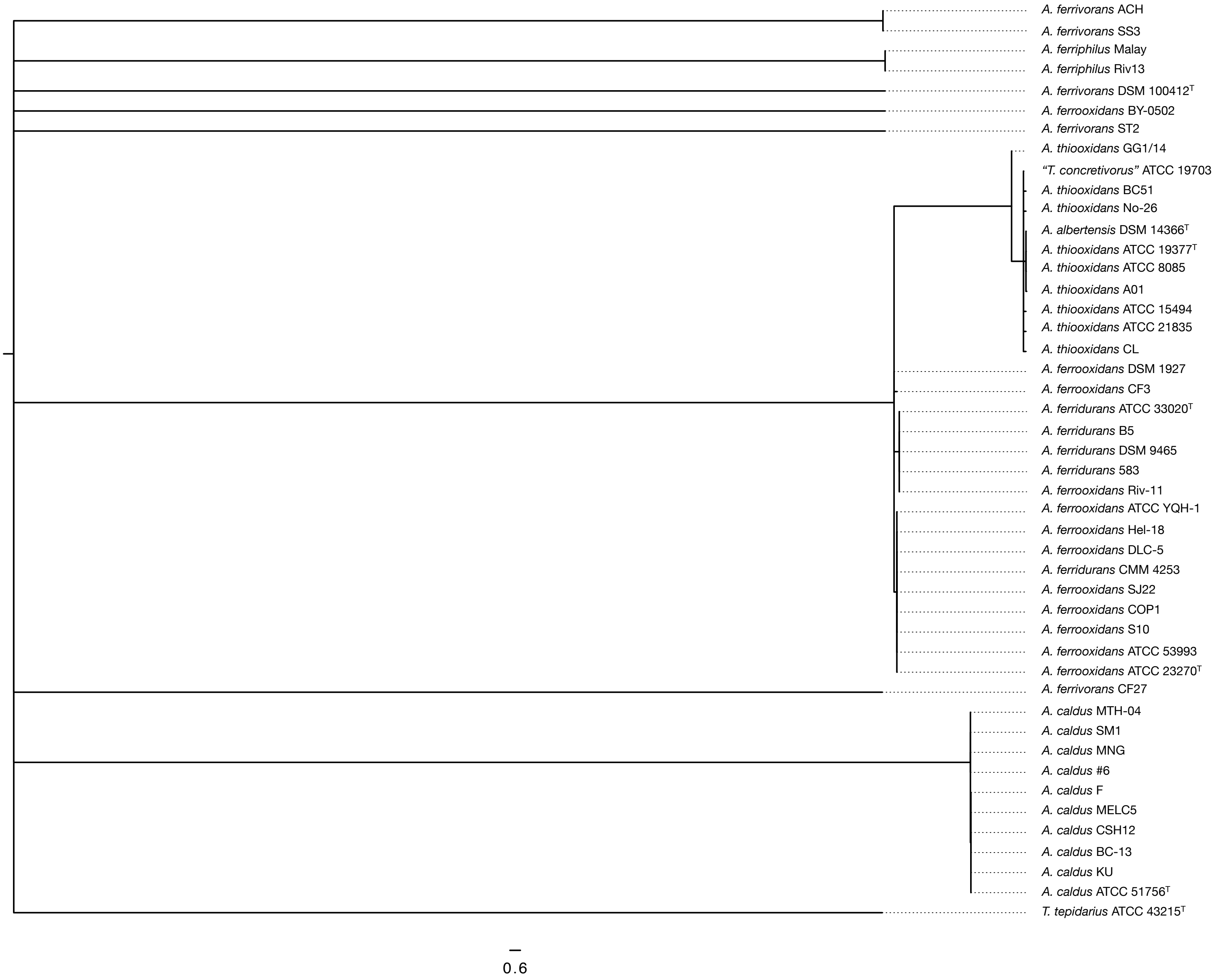
A. ferrivorans ACH
A. ferrivorans SS3
A. ferriphilus Malay
A. ferriphilus Riv13
A. ferrivorans DSM 100412T
A. ferrooxidans BY-0502
A. ferrivorans ST2
A. thiooxidans GG1/14
"T. concretivorus" ATCC 19703
A. thiooxidans BC51
A. thiooxidans No-26
A. albertensis DSM 14366T
A. thiooxidans ATCC 19377T
A. thiooxidans ATCC 8085
A. thiooxidans A01
A. thiooxidans ATCC 15494
A. thiooxidans ATCC 21835
A. thiooxidans CL
A. ferrooxidans DSM 1927
A. ferrooxidans CF3
A. ferridurans ATCC 33020T
A. ferridurans B5
A. ferridurans DSM 9465
A. ferridurans 583
A. ferrooxidans Riv-11
A. ferrooxidans ATCC YQH-1
A. ferrooxidans Hel-18
A. ferrooxidans DLC-5
A. ferridurans CMM 4253
A. ferrooxidans SJ22
A. ferrooxidans COP1
A. ferrooxidans S10
A. ferrooxidans ATCC 53993
A. ferrooxidans ATCC 23270T
A. ferrivorans CF27
A. caldus MTH-04
A. caldus SM1
A. caldus MNG
A. caldus #6
A. caldus F
A. caldus MELC5
A. caldus CSH12
A. caldus BC-13
A. caldus KU
A. caldus ATCC 51756T
T. tepidarius ATCC 43215T
0.6

K)

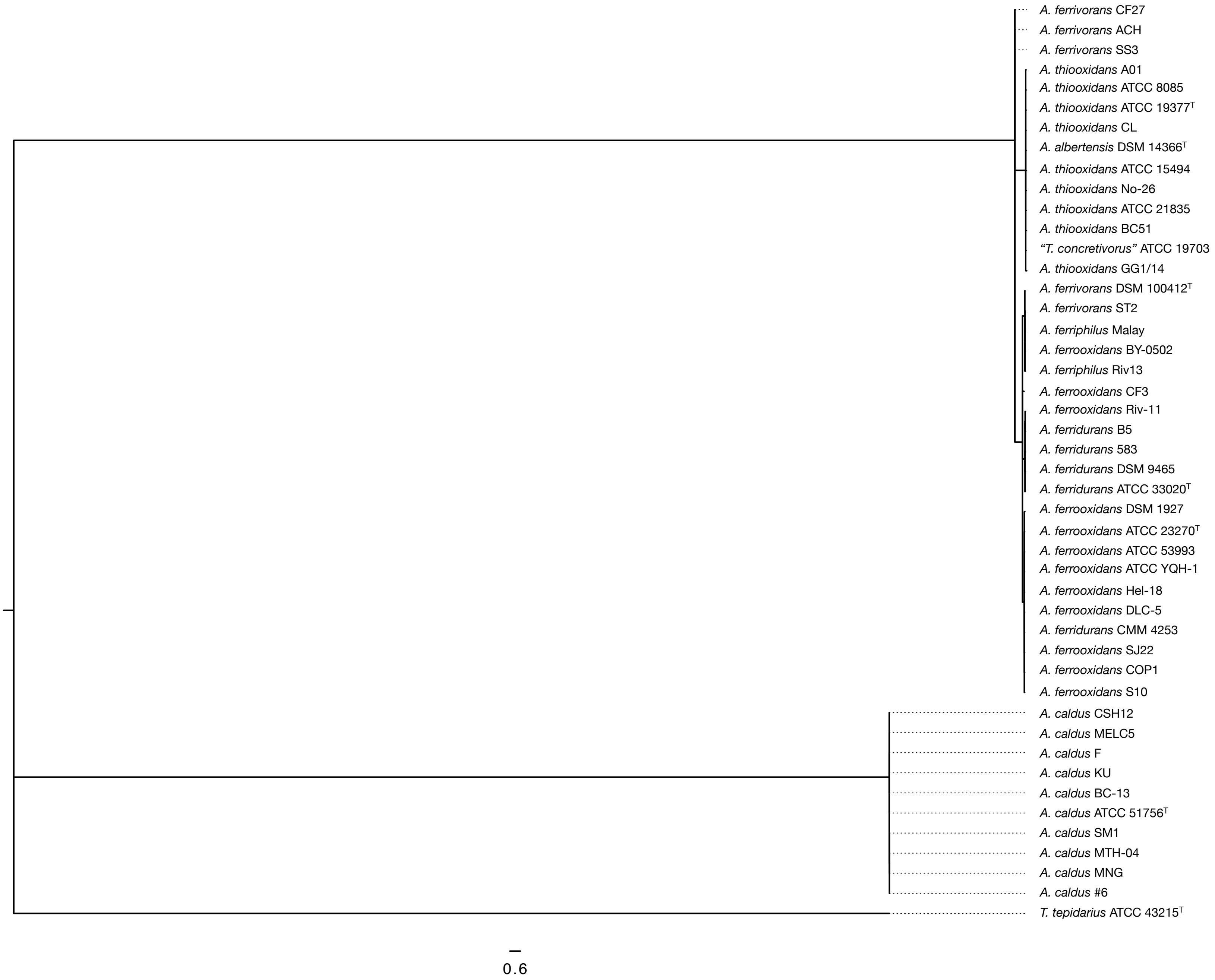
A. ferrivorans CF27
A. ferrivorans ACH
A. ferrivorans SS3
A. thiooxidans A01
A. thiooxidans ATCC 8085
A. thiooxidans ATCC 19377T
A. thiooxidans CL
A. albertensis DSM 14366T
A. thiooxidans ATCC 15494
A. thiooxidans No-26
A. thiooxidans ATCC 21835
A. thiooxidans BC51
"T. concretivorus" ATCC 19703
A. thiooxidans GG1/14
A. ferrivorans DSM 100412T
A. ferrivorans ST2
A. ferriphilus Malay
A. ferrooxidans BY-0502
A. ferriphilus Riv13
A. ferrooxidans CF3
A. ferrooxidans Riv-11
A. ferridurans B5
A. ferridurans 583
A. ferridurans DSM 9465
A. ferridurans ATCC 33020T
A. ferrooxidans DSM 1927
A. ferrooxidans ATCC 23270T
A. ferrooxidans ATCC 53993
A. ferrooxidans ATCC YQH-1
A. ferrooxidans Hel-18
A. ferrooxidans DLC-5
A. ferridurans CMM 4253
A. ferrooxidans SJ22
A. ferrooxidans COP1
A. ferrooxidans S10
A. caldus CSH12
A. caldus MELC5
A. caldus F
A. caldus KU
A. caldus BC-13
A. caldus ATCC 51756T
A. caldus SM1
A. caldus MTH-04
A. caldus MNG
A. caldus #6
T. tepidarius ATCC 43215T
0.6

L)

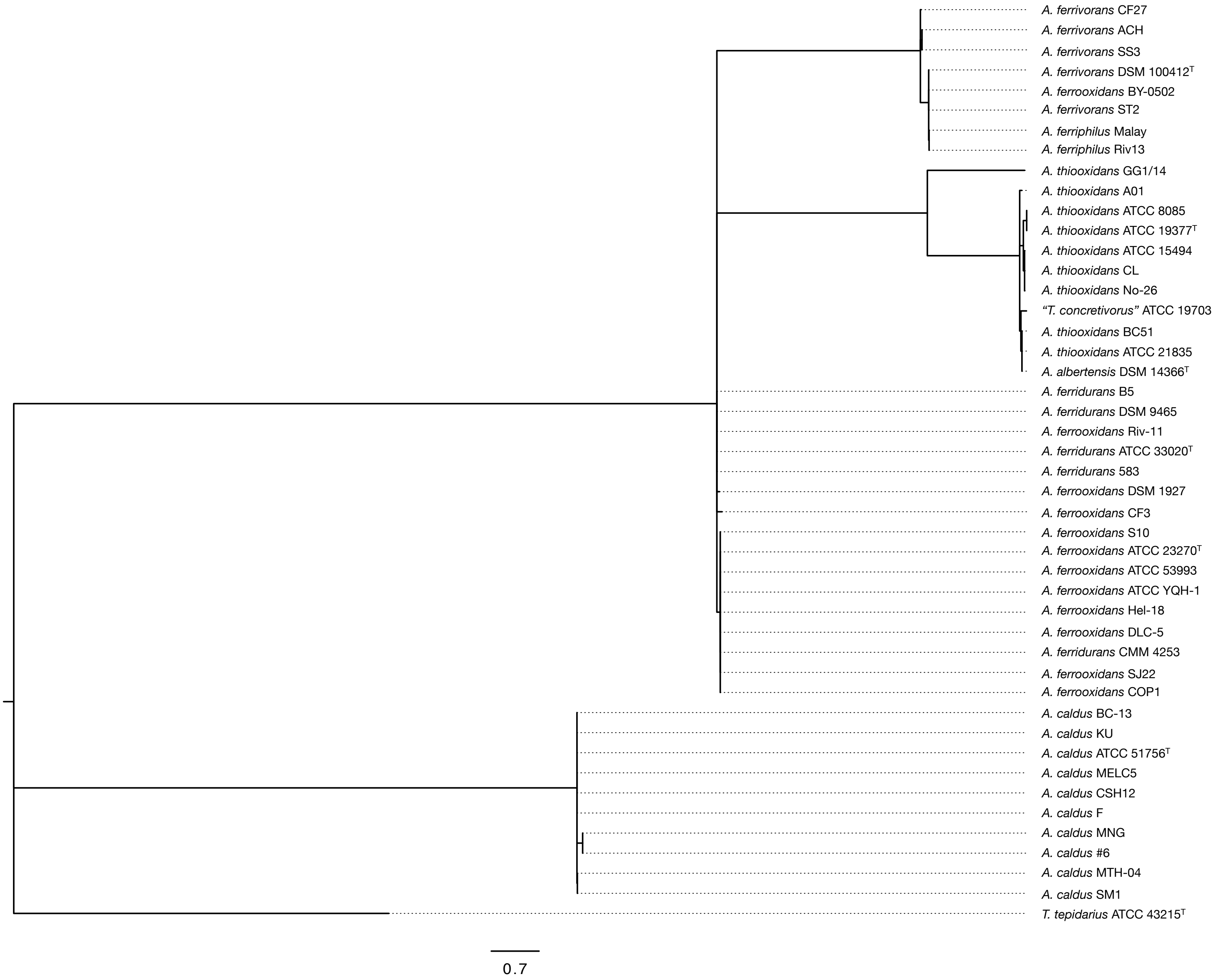
A. ferrivorans CF27
A. ferrivorans ACH
A. ferrivorans SS3
A. ferrivorans DSM 100412T
A. ferrooxidans BY-0502
A. ferrivorans ST2
A. ferriphilus Malay
A. ferriphilus Riv13
A. thiooxidans GG1/14
A. thiooxidans A01
A. thiooxidans ATCC 8085
A. thiooxidans ATCC 19377T
A. thiooxidans ATCC 15494
A. thiooxidans CL
A. thiooxidans No-26
"T. concretivorus" ATCC 19703
A. thiooxidans BC51
A. thiooxidans ATCC 21835
A. albertensis DSM 14366T
A. ferridurans B5
A. ferridurans DSM 9465
A. ferrooxidans Riv-11
A. ferridurans ATCC 33020T
A. ferridurans 583
A. ferrooxidans DSM 1927
A. ferrooxidans CF3
A. ferrooxidans S10
A. ferrooxidans ATCC 23270T
A. ferrooxidans ATCC 53993
A. ferrooxidans ATCC YQH-1
A. ferrooxidans Hel-18
A. ferrooxidans DLC-5
A. ferridurans CMM 4253
A. ferrooxidans SJ22
A. ferrooxidans COP1
A. caldus BC-13
A. caldus KU
A. caldus ATCC 51756T
A. caldus MELC5
A. caldus CSH12
A. caldus F
A. caldus MNG
A. caldus #6
A. caldus MTH-04
A. caldus SM1
T. tepidarius ATCC 43215T
0.7

M)

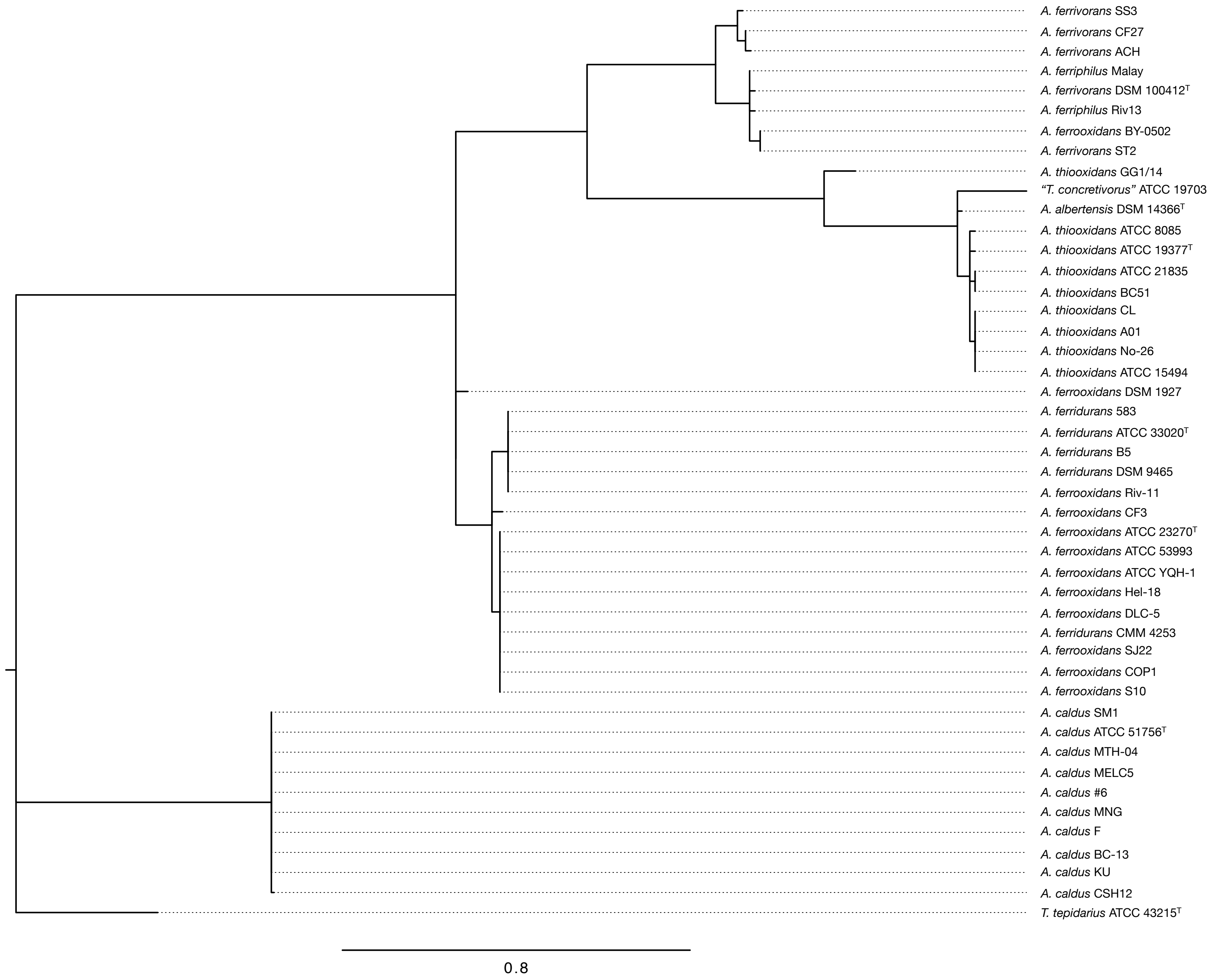
A. ferrivorans SS3
A. ferrivorans CF27
A. ferrivorans ACH
A. ferriphilus Malay
A. ferrivorans DSM 100412T
A. ferriphilus Riv13
A. ferrooxidans BY-0502
A. ferrivorans ST2
A. thiooxidans GG1/14
"T. concretivorus" ATCC 19703
A. albertensis DSM 14366T
A. thiooxidans ATCC 8085
A. thiooxidans ATCC 19377T
A. thiooxidans ATCC 21835
A. thiooxidans BC51
A. thiooxidans CL
A. thiooxidans A01
A. thiooxidans No-26
A. thiooxidans ATCC 15494
A. ferrooxidans DSM 1927
A. ferridurans 583
A. ferridurans ATCC 33020T
A. ferridurans B5
A. ferridurans DSM 9465
A. ferrooxidans Riv-11
A. ferrooxidans CF3
A. ferrooxidans ATCC 23270T
A. ferrooxidans ATCC 53993
A. ferrooxidans ATCC YQH-1
A. ferrooxidans Hel-18
A. ferrooxidans DLC-5
A. ferridurans CMM 4253
A. ferrooxidans SJ22
A. ferrooxidans COP1
A. ferrooxidans S10
A. caldus SM1
A. caldus ATCC 51756T
A. caldus MTH-04
A. caldus MELC5
A. caldus #6
A. caldus MNG
A. caldus F
A. caldus BC-13
A. caldus KU
A. caldus CSH12
T. tepidarius ATCC 43215T
0.8

N)

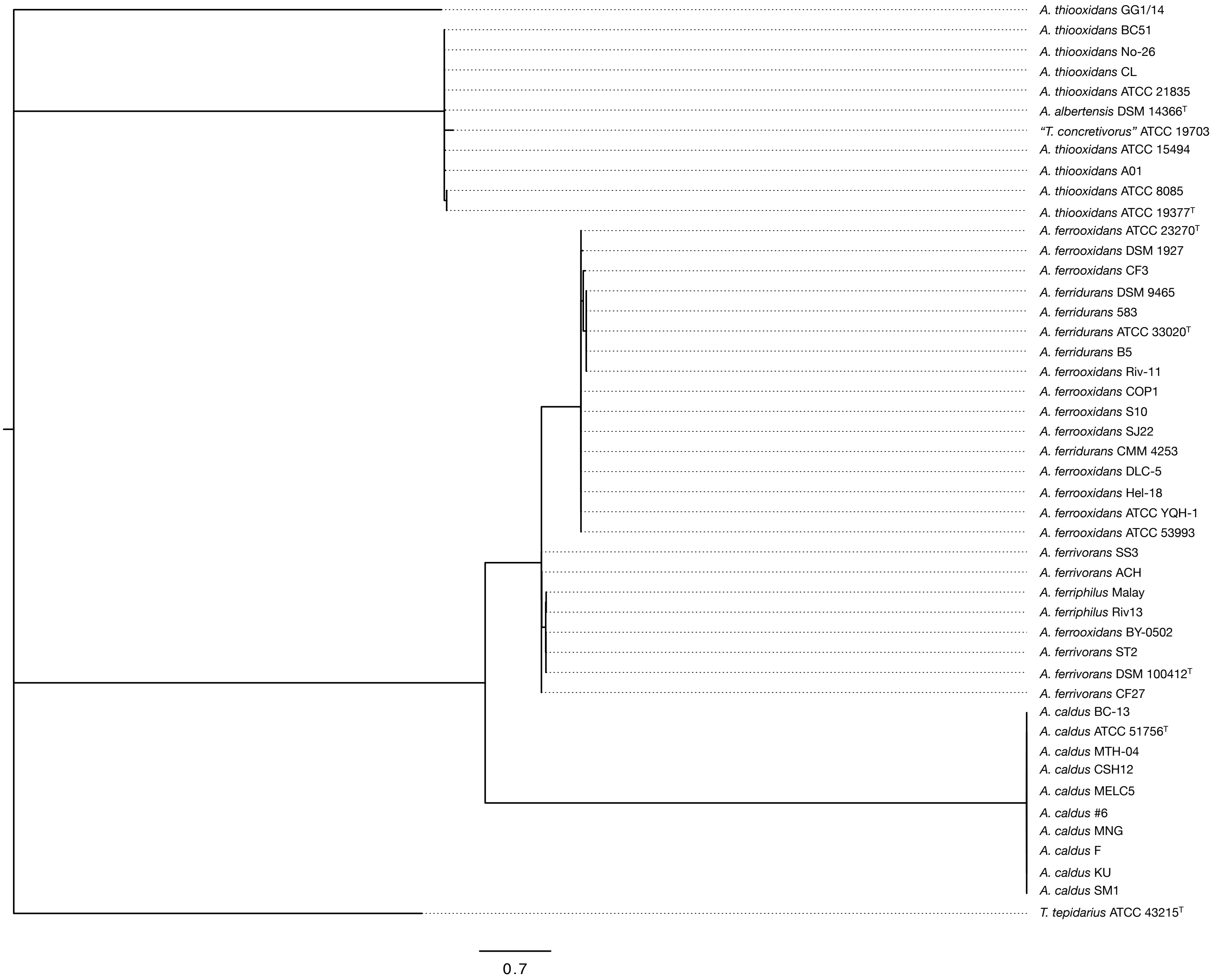

A. thiooxidans GG1/14
A. thiooxidans BC51
A. thiooxidans No-26
A. thiooxidans CL
A. thiooxidans ATCC 21835
A. albertensis DSM 14366T
"T. concretivorus" ATCC 19703
A. thiooxidans ATCC 15494
A. thiooxidans A01
A. thiooxidans ATCC 8085
A. thiooxidans ATCC 19377T
A. ferrooxidans ATCC 23270T
A. ferrooxidans DSM 1927
A. ferrooxidans CF3
A. ferridurans DSM 9465
A. ferridurans 583
A. ferridurans ATCC 33020T
A. ferridurans B5
A. ferrooxidans Riv-11
A. ferrooxidans COP1
A. ferrooxidans S10
A. ferrooxidans SJ22
A. ferridurans CMM 4253
A. ferrooxidans DLC-5
A. ferrooxidans Hel-18
A. ferrooxidans ATCC YQH-1
A. ferrooxidans ATCC 53993
A. ferrivorans SS3
A. ferrivorans ACH
A. ferriphilus Malay
A. ferriphilus Riv13
A. ferrooxidans BY-0502
A. ferrivorans ST2
A. ferrivorans DSM 100412T
A. ferrivorans CF27
A. caldus BC-13
A. caldus ATCC 51756T
A. caldus MTH-04
A. caldus CSH12
A. caldus MELC5
A. caldus #6
A. caldus MNG
A. caldus F
A. caldus KU
A. caldus SM1
T. tepidarius ATCC 43215T
0.7

O)

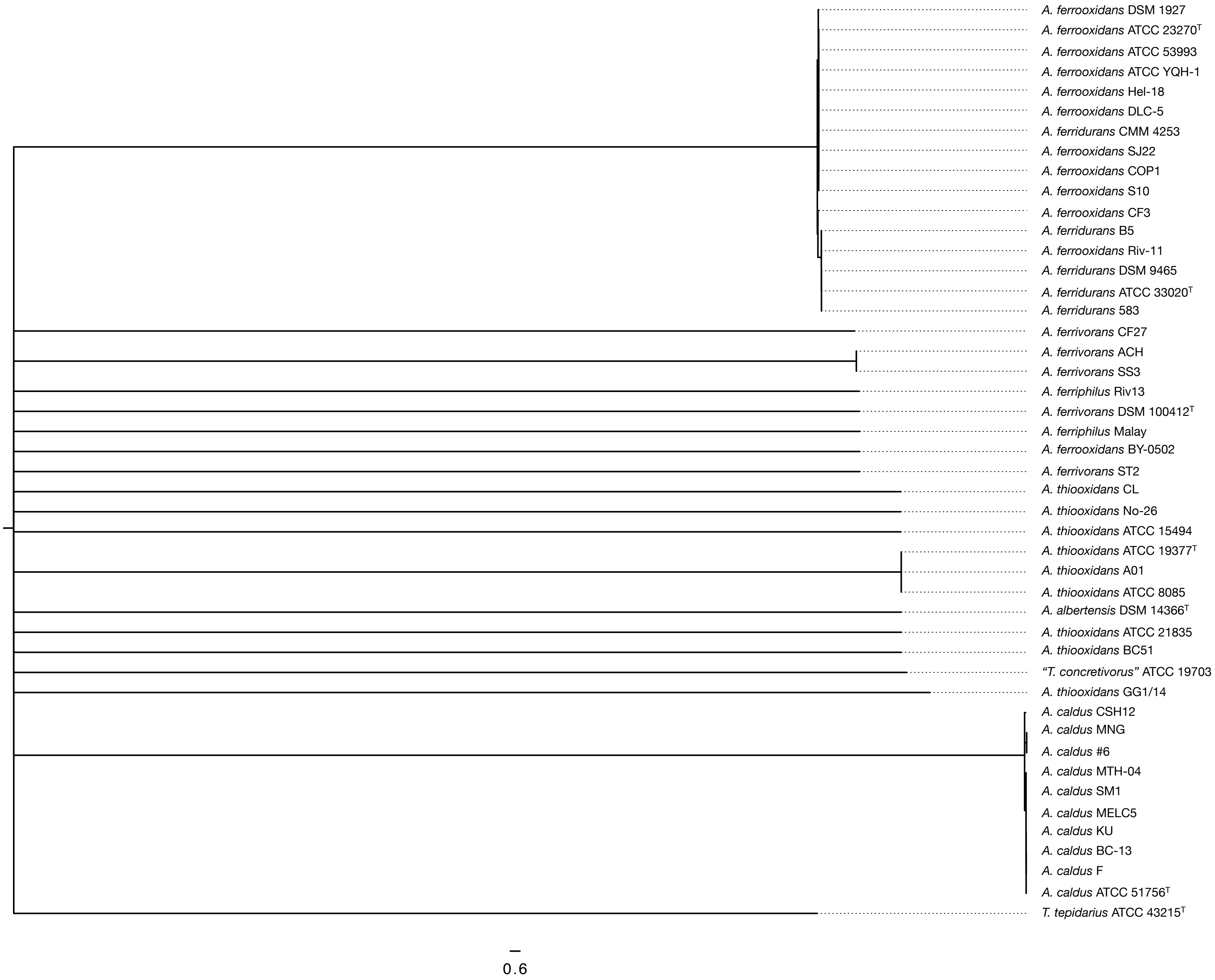

A. ferrooxidans DSM 1927
A. ferrooxidans ATCC 23270T
A. ferrooxidans ATCC 53993
A. ferrooxidans ATCC YQH-1
A. ferrooxidans Hel-18
A. ferrooxidans DLC-5
A. ferridurans CMM 4253
A. ferrooxidans SJ22
A. ferrooxidans COP1
A. ferrooxidans S10
A. ferrooxidans CF3
A. ferridurans B5
A. ferrooxidans Riv-11
A. ferridurans DSM 9465
A. ferridurans ATCC 33020T
A. ferridurans 583
A. ferrivorans CF27
A. ferrivorans ACH
A. ferrivorans SS3
A. ferriphilus Riv13
A. ferrivorans DSM 100412T
A. ferriphilus Malay
A. ferrooxidans BY-0502
A. ferrivorans ST2
A. thiooxidans CL
A. thiooxidans No-26
A. thiooxidans ATCC 15494
A. thiooxidans ATCC 19377T
A. thiooxidans A01
A. thiooxidans ATCC 8085
A. albertensis DSM 14366T
A. thiooxidans ATCC 21835
A. thiooxidans BC51
"T. concretivorus" ATCC 19703
A. thiooxidans GG1/14
A. caldus CSH12
A. caldus MNG
A. caldus #6
A. caldus MTH-04
A. caldus SM1
A. caldus MELC5
A. caldus KU
A. caldus BC-13
A. caldus F
A. caldus ATCC 51756T
T. tepidarius ATCC 43215T
0.6

P)

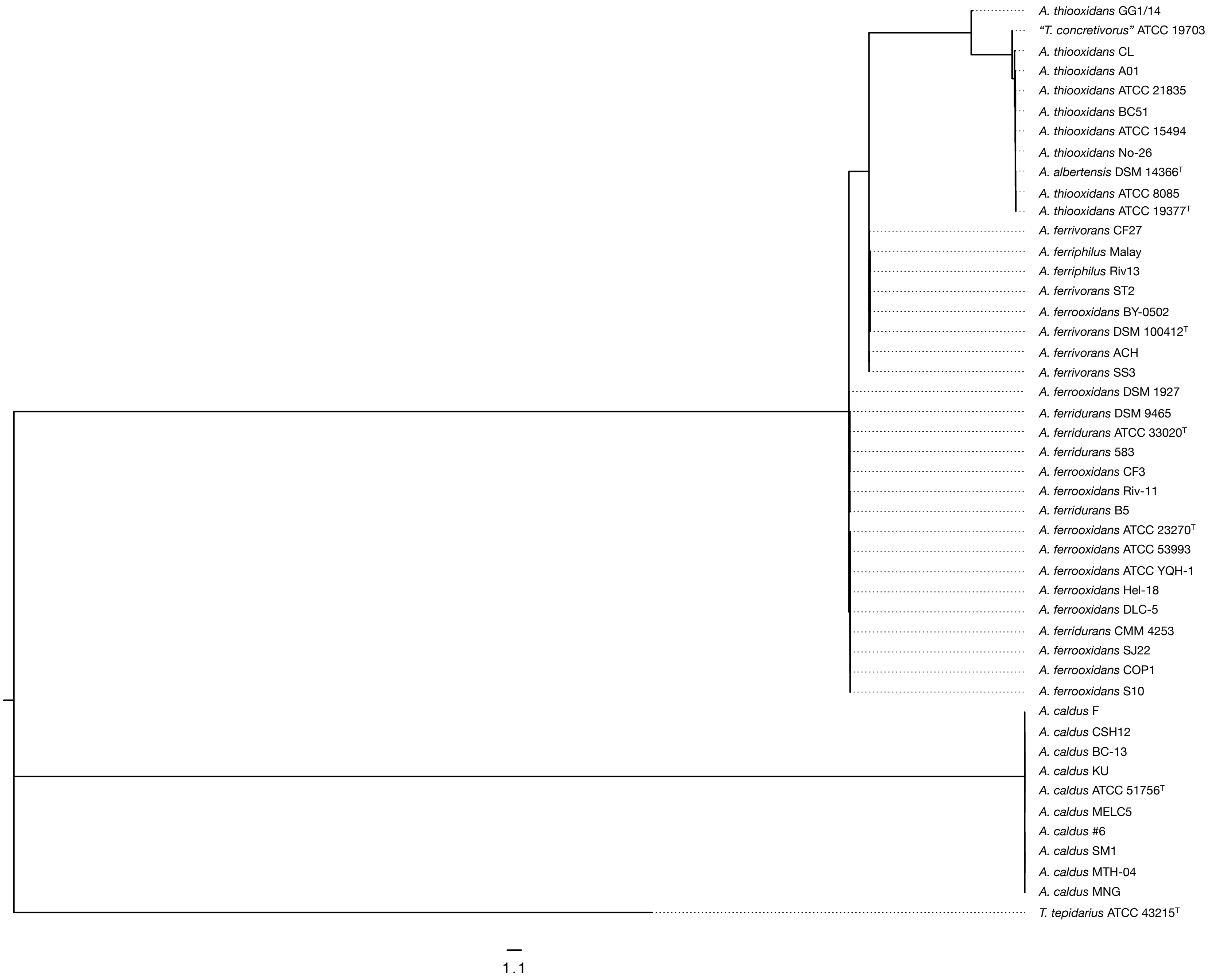
A. thiooxidans GG1/14
"T. concretivorus" ATCC 19703
A. thiooxidans CL
A. thiooxidans A01
A. thiooxidans ATCC 21835
A. thiooxidans BC51
A. thiooxidans ATCC 15494
A. thiooxidans No-26
A. albertensis DSM 14366T
A. thiooxidans ATCC 8085
A. thiooxidans ATCC 19377T
A. ferrivorans CF27
A. ferriphilus Malay
A. ferriphilus Riv13
A. ferrivorans ST2
A. ferrooxidans BY-0502
A. ferrivorans DSM 100412T
A. ferrivorans ACH
A. ferrivorans SS3
A. ferrooxidans DSM 1927
A. ferridurans DSM 9465
A. ferridurans ATCC 33020T
A. ferridurans 583
A. ferrooxidans CF3
A. ferrooxidans Riv-11
A. ferridurans B5
A. ferrooxidans ATCC 23270T
A. ferrooxidans ATCC 53993
A. ferrooxidans ATCC YQH-1
A. ferrooxidans Hel-18
A. ferrooxidans DLC-5
A. ferridurans CMM 4253
A. ferrooxidans SJ22
A. ferrooxidans COP1
A. ferrooxidans S10
A. caldus F
A. caldus CSH12
A. caldus BC-13
A. caldus KU
A. caldus ATCC 51756T
A. caldus MELC5
A. caldus #6
A. caldus SM1
A. caldus MTH-04
A. caldus MNG
T. tepidarius ATCC 43215T
1.1

Supplementary Figure 5. Individual MLSA marker gene phylogenentic trees for 45 *Acidithiobacillus* strains representative of available subclades undercovered in the 16S rRNA analysis built using maximum likelihood. Numbers at the nodes indicate the bootstrap values of 1,000 replicates (%). Numbers on the individual branches indicate dN/dS values greater than 1.0) The bar represents the expected nucleotide substitutions per site. Trees represented as cladograms (**A**) *ihfß*, (**B**) *trx*, (**C**) *rplU*, (**D**) *hslV*, (**E**) *fdx*, (**F**) *ilvE*, (**G**) *rpsE*, (**H**) *ruvB*. Trees represented as phylograms **(I)** *ihfß*, (**J**) *trx*, (**K**) *rplU*, (**L**) *hslV*, (**M**) *fdx*, (**N**) *ilvE*, (**O**) *rpsE*, (**P**) *ruvB*.
